# Cell Division Protein FtsZ Is Unfolded for N-Terminal Degradation by Antibiotic-Activated ClpP

**DOI:** 10.1128/mBio.01006-20

**Published:** 2020-06-30

**Authors:** Nadine Silber, Stefan Pan, Sina Schäkermann, Christian Mayer, Heike Brötz-Oesterhelt, Peter Sass

**Affiliations:** a Department of Microbial Bioactive Compounds, Interfaculty Institute of Microbiology and Infection Medicine, University of Tuebingen, Tuebingen, Germany; b Applied Microbiology, Ruhr University Bochum, Bochum, Germany; c Cluster of Excellence-Controlling Microbes to Fight Infections, Tuebingen, Germany; Max Planck Institute for Terrestrial Microbiology

**Keywords:** ADEP, antibiotics, acyldepsipeptides, guanosine nucleotides, protein unfolding, cytokinesis, Clp protease, Clp-ATPases

## Abstract

Antibiotic acyldepsipeptides (ADEPs) deregulate ClpP, the proteolytic core of the bacterial Clp protease, thereby inhibiting its native functions and concomitantly activating it for uncontrolled proteolysis of nonnative substrates. Importantly, although ADEP-activated ClpP is assumed to target multiple polypeptide and protein substrates in the bacterial cell, not all proteins seem equally susceptible. In Bacillus subtilis, the cell division protein FtsZ emerged to be particularly sensitive to degradation by ADEP-activated ClpP at low inhibitory ADEP concentrations. In fact, FtsZ is the only bacterial protein that has been confirmed to be degraded *in vitro* as well as within bacterial cells so far. However, the molecular reason for this preferred degradation remained elusive. Here, we report the unexpected finding that ADEP-activated ClpP alone, in the absence of any Clp-ATPase, leads to an unfolding and subsequent degradation of the N-terminal domain of FtsZ, which can be prevented by the stabilization of the FtsZ fold via nucleotide binding. At elevated antibiotic concentrations, importantly, the C terminus of FtsZ is notably targeted for degradation in addition to the N terminus. Our results show that different target structures are more or less accessible to ClpP, depending on the ADEP level present. Moreover, our data assign a Clp-ATPase-independent protein unfolding capability to the ClpP core of the bacterial Clp protease and suggest that the protein fold of FtsZ may be more flexible than previously anticipated.

## Abstract (precis)

Acyldepsipeptide (ADEP) antibiotics effectively kill multidrug-resistant Gram-positive pathogens, including vancomycin-resistant enterococcus, penicillin-resistant Streptococcus pneumoniae (PRSP), and methicillin-resistant Staphylococcus aureus (MRSA). The antibacterial activity of ADEP depends on a new mechanism of action, i.e., the deregulation of bacterial protease ClpP that leads to bacterial self-digestion. Our data allow new insights into the mode of ADEP action by providing a molecular explanation for the distinct bacterial phenotypes observed at low versus high ADEP concentrations. In addition, we show that ClpP alone, in the absence of any unfoldase or energy-consuming system, and only activated by the small molecule antibiotic ADEP, leads to the unfolding of the cell division protein FtsZ.

## INTRODUCTION

Antibiotic acyldepsipeptides (ADEPs) kill Gram-positive bacteria, including human pathogens, by deregulating the bacterial protease Clp (1–4). The Clp protease is conserved across most bacterial species, where it is naturally involved in protein homeostasis and regulatory proteolysis. Clp is important for maintaining vital cellular functions particularly under stress conditions and directs developmental processes like cell differentiation, genetic competence, and virulence (5, 6). Thus, Clp protease has emerged as a novel target for antibiotic action and virulence inhibition (7–9).

Clp protease constitutes a macromolecular complex that is composed of a proteolytic core, ClpP, that associates with specific hexameric unfoldases, the Clp-ATPases, and accessory adaptor proteins (8, 10). ClpP forms a tetradecameric, barrel-shaped structure with the proteolytic chamber secluded within the assembled complex. The cognate Clp-ATPases interact with the ClpP tetradecamer via distinct hydrophobic pockets at both sides of the barrel (11). Substrate access to the proteolytic chamber is strictly controlled by the partner Clp-ATPases, which recognize the respective protein substrates, unfold them in an ATP-dependent manner, and thread the unfolded polypeptide chains through the apical and distal entrance pores of the ClpP barrel (12). It is important to note that ClpP is capable of degrading peptides on its own, but not fully folded proteins, and strictly depends on ATP-driven Clp-ATPases for proteolytic activation in the natural context (10, 12).

ADEP antibiotics compete with the Clp-ATPases for the same binding sites on ClpP, which results in a dual mechanism of action; by binding to the hydrophobic pockets of ClpP, ADEPs abrogate the interaction of ClpP with its partner Clp-ATPases, thereby preventing all cellular functions of Clp in general and regulatory proteolysis (13). This is sufficient to kill mycobacteria (14, 15), which rely on a functional Clp system for viability (16). However, in most bacterial species, including Bacillus subtilis or Staphylococcus aureus, Clp is not essential for growth, and thus, mere Clp inhibition is not sufficient to kill bacteria under normal growth conditions. In such species, a ClpP activation mechanism leads to bacterial killing; ADEP binding supports the oligomerization process of ClpP (13) and locks the catalytic triads in an active conformation via allosteric conformational control (17, 18). ADEP additionally triggers a closed- to open-gate structural transition of the N-terminal segments of ClpP that opens the substrate entrance pore to the proteolytic chamber (19, 20), which is otherwise tightly closed (21). As a result, nonnative polypeptides and protein substrates are now allowed to enter the proteolytic chamber of ClpP and are subsequently degraded in a Clp-ATPase-independent manner (1, 13).

Depending on the applied ADEP concentration, treated bacteria show distinct phenotypes. At high ADEP concentrations, several times the MIC, cells rapidly stop growing due to ceased biomass production (22, 23). This phenotype may be readily explained by the broad destructive capacity of ClpP activation, and we showed earlier that ADEP activates ClpP to degrade nascent protein chains during translation (13), likely causing a depletion of several essential bacterial proteins. In contrast, at concentrations very close to the MIC, ADEP exposure results in a more specific phenotype, namely, bacterial cells retain considerable biosynthetic capacity, with macromolecular syntheses and biomass production proceeding for hours (22). However, cell size significantly increases, as indicated by the swelling of coccoid S. aureus and Streptococcus pneumoniae cells as well as an impressive filamentation of rod-shaped B. subtilis, eventually leading to cell death (22, 23). A strong ongoing biosynthetic capacity with blocked cytokinesis points to a preferential proteolytic target in bacterial cell division, and we reported earlier that this is due to the untimely degradation of the essential cell division protein FtsZ by ADEP-activated ClpP (22). This prominent phenotype clearly distinguishes FtsZ as being particularly susceptible to ADEP-ClpP. Intriguingly, the B. subtilis FtsZ protein was completely and easily degraded by ADEP-ClpP *in vitro*, while several other folded proteins were not (22). In exploring this enigma, it emerged that the short, hydrophobic N terminus of B. subtilis FtsZ is particularly prone to be targeted by ADEP-ClpP, leading to the unfolding of the FtsZ protein over the course of the degradation process, while degradation of the extended and flexible C terminus of FtsZ is only triggered at increased ADEP concentration. Thereby, our results allow new insight into the stability of the FtsZ protein, suggest extending the mechanistic capabilities of ADEP-ClpP toward ATP-independent protein unfolding, and provide a molecular explanation for the different bacterial phenotypes observed at low versus high ADEP concentrations.

## RESULTS

### ADEP-activated ClpP preferentially targets the short N terminus of FtsZ.

**FIG 1:**
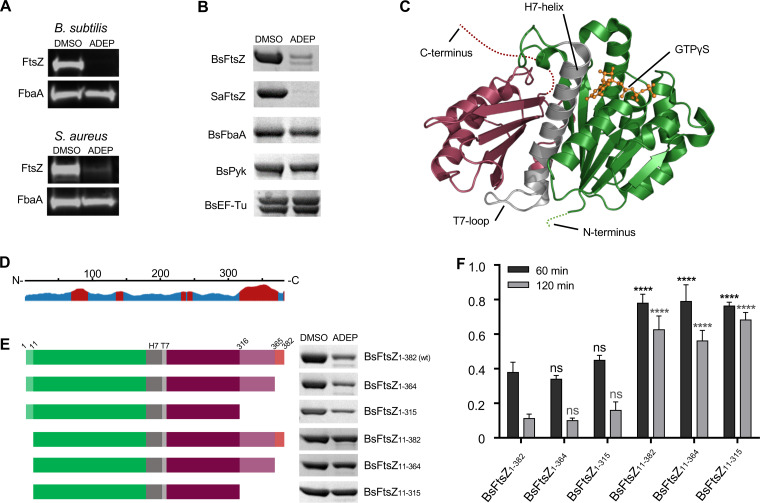
ADEP-activated ClpP preferentially targets the N terminus of FtsZ. (A) Immunoblotting of B. subtilis 168 and S. aureus NCTC8325 cells that were treated with ADEP concentrations close to the MIC (0.25 μg/ml ADEP2 for B. subtilis 168 and 1 μg/ml ADEP2 for S. aureus NCTC 8325), a concentration that leads to a pronounced filamentation phenotype of B. subtilis and swelling of S. aureus. Here, immunoblots show the rapid degradation of FtsZ compared with the untreated control (DMSO). In contrast, the abundance of FbaA was unaltered under the same conditions. Immunodetection of FtsZ and FbaA was performed using specific anti-FtsZ and anti-FbaA antibodies, respectively. Samples were taken after 120 min. DMSO was used as a control. (B) *In vitro* degradation assays using BsFtsZ and S. aureus FtsZ (SaFtsZ) as well as B. subtilis FbaA (BsFbaA), elongation factor Tu (BsEF-Tu), and pyruvate kinase (BsPyk) as purified protein substrates for ADEP-activated ClpP. In these assays, low concentrations of ADEP and ClpP were applied (1.5 μM ClpP; 1.5 μM ADEP). Samples were taken after 60 min. DMSO was used as a control. Of note, phosphorylation of BsEF-Tu often produces a doublet visible in SDS-PAGE, most probably resulting from partial phosphorylation of the multiply phosphorylated substrate (65). All experiments were performed at least in triplicate; representative images are depicted. (C) The crystal structure of B. subtilis FtsZ (BsFtsZ; Protein Data Bank entry 2RHO) bound to the nonhydrolyzable GTP-analog GTPγS shows two independently folding domains (24–26), the N-terminal domain harboring the nucleotide binding site (in green) as well as the C-terminal domain (in currant red). GTPγS is indicated in orange. Both domains are connected via the central H7 helix and the T7 loop (in gray). The short N terminus (amino acids 1 to 10) and the flexible C terminus (amino acids 316 to 382) are indicated by dotted lines. (D) Disorder prediction of BsFtsZ was computed with JRONN software (66) (Protein Data Bank entry 2RHO; https://www.rcsb.org). Predicted disordered regions (in red) and ordered regions (in blue) are indicated. (E) The left image panel provides a schematic representation of N- and C-terminal protein truncations of FtsZ used in degradation assays aligned to the disorder plot in D. The uppermost protein represents full-length FtsZ. The right image panel shows ADEP-ClpP degradation assays of full-length and truncated BsFtsZ proteins (aligned to the corresponding schematic on the left). In these assays, low concentrations of ADEP and ClpP were used (1.5 μM ClpP; 1.5 μM ADEP). Samples were taken after 60 min; DMSO was used as a control. (F) Densitometry of protein bands from SDS-PAGE was performed to compare FtsZ protein amounts in ADEP-treated samples with the corresponding DMSO control reactions after 60 min (black bars) and 120 min (gray bars). The respective DMSO control was set to 1.0 (100%). For each FtsZ variant, the data were collected from three different degradation assays and SDS-PAGE analyses using a standard curve. Protein amounts were calculated using a standard FtsZ concentration series that was applied on each SDS-PAGE. The plotted data depict the corresponding mean values, with standard deviations indicated by error bars. Compared with BsFtsZ_1-382_, there was no significant (ns) difference regarding the remaining protein amount of the C-terminally truncated FtsZ mutant proteins BsFtsZ_1-364_ and _Bs_FtsZ_1-315_ after 60 or 120 min of incubation with ADEP-ClpP. Contrariwise, the N-terminally truncated FtsZ proteins BsFtsZ_11-382_, BsFtsZ_11-364_, and BsFtsZ_11-315_ were characterized by significantly less degradation of the FtsZ protein by ADEP-ClpP (****, *P* < 0.0001) than BsFtsZ_1-382_. For all assays with substrates originating from B. subtilis or S. aureus, we used the corresponding BsClpP or S. aureus ClpP (SaClpP) proteins, respectively, with the according activity buffers (see Materials and Methods section). All results were confirmed by at least three independent experiments, and images of representative experiments are depicted.

In this study, we set out to determine the molecular basis for the observed sensitivity of B. subtilis FtsZ toward ADEP-ClpP. To validate FtsZ as a preferred target for ADEP-ClpP, we first compared the abundance of the fructose-bisphosphate aldolase (FbaA) in relation to FtsZ in B. subtilis and S. aureus cells that were exposed to ADEP concentrations close to the MIC (2× to 3× MIC, i.e., 0.25 μg/ml ADEP2 for B. subtilis 168 and 1 μg/ml ADEP2 for S. aureus NCTC 8325) (Fig. 1A). Fragments of FbaA (as well as of elongation factor Tu [EF-Tu] and pyruvate kinase [Pyk]) were detected in notable amounts in a previous proteomics study of S. aureus cells exposed to ADEP at elevated concentrations (10× MIC) for an extended time period (3). Therefore, FbaA was assumed to be a target for ADEP-activated ClpP in this previous study. Using immunodetection of FtsZ and FbaA proteins, our results confirmed the rapid and complete degradation of FtsZ after 120 min in ADEP-treated cells; however, the protein concentration of FbaA remained unaltered under these conditions (Fig. 1A). To study this aspect in more detail, we proceeded to test the degradation of B. subtilis FbaA, EF-Tu, and Pyk (BsFbaA, BsEF-Tu, and BsPyk, respectively) by ADEP-ClpP using purified proteins *in vitro*. Here, BsFbaA, BsEF-Tu, BsPyk were not notably degraded by ADEP-ClpP under the same conditions *in vitro*, in contrast to FtsZ (Fig. 1B), thereby clearly corroborating a preferential degradation of FtsZ by ADEP-ClpP.

Next, we looked into the published crystal structure of FtsZ to identify structural features that may be potentially prone to degradation. B. subtilis FtsZ (BsFtsZ; Protein Data Bank entry 2RHO) (Fig. 1C) comprises an overall globular core protein with two independently folding domains (24–26), the N-terminal and the C-terminal domain, which are connected via the central core helix H7 followed by the T7 loop. The N-terminal domain harbors the nucleotide binding site which interacts with the T7 loop of a second FtsZ monomer to form the GTPase catalytic site (27). The long flexible C terminus (amino acids 316 to 382), which contains the interaction interface for other cell division proteins, as well as the short N terminus (amino acids 1 to 10), are not resolved in the crystal structure due to their inherent flexibility. *In silico* disorder predictions visualized the overall structural disorder of the C terminus (Fig. 1D), making it a structural feature likely to be attacked by ADEP-ClpP, as this flexible amino acid tail may easily diffuse through the opened entrance pores into the degradation chamber of ADEP-ClpP. To test this hypothesis, we constructed several mutant proteins of BsFtsZ and tested them in *in vitro* ADEP-ClpP degradation assays (Fig. 1E and F; see Fig. S1 in the supplemental material). Surprisingly, FtsZ mutants with deleted C termini, either short truncations omitting the last 18 amino acids containing the interaction interface (BsFtsZ_1-364_) as well as longer truncations deleting the complete flexible C terminus (BsFtsZ_1-315_), were still degraded similarly to full-length BsFtsZ_1-382_. However, degradation was significantly impaired when we deleted the short N terminus of FtsZ (BsFtsZ_11-382_, BsFtsZ_11-364_, and BsFtsZ_11-315_), identifying this structural feature as the preferred target site for ADEP-ClpP. Of note, all tested FtsZ protein variants were functional as shown in GTPase assays (see Fig. S2 in the supplemental material). We selected the synthetic derivative ADEP2 for all degradation assays in this study because, among a small series of ADEP congeners tested, it proved particularly effective at activating B. subtilis ClpP (BsClpP) for the degradation of BsFtsZ (see Fig. S3 in the supplemental material) and we had also used ADEP2 for degradation assays in our previous studies (14, 22). We further incubated FtsZ either alone or with ADEP (both in the absence of ClpP) for 60 min at 37°C, which did not affect FtsZ protein stability or functionality (see Fig. S4 in the supplemental material), ruling out off-target effects of ADEP on FtsZ or denaturing of FtsZ over the course of the *in vitro* assay.

10.1128/mBio.01006-20.1 FIG S1 Time course of the degradation of FtsZ wild-type and mutant proteins by ADEP-ClpP. Degradation of FtsZ wild-type and mutant proteins using low concentrations of ADEP and ClpP (1.5 μM ClpP; 1.5 μM ADEP) was followed over time. Samples were taken every 10 min over a time period of 60 min. DMSO was used as a control. The intensity of the ClpP protein band also serves as a loading control. Download FIG S1, PDF file, 0.6 MB. Copyright © 2020 Silber et al. 2020 Silber et al. This content is distributed under the terms of the Creative Commons Attribution 4.0 International license.

10.1128/mBio.01006-20.2 FIG S2 GTPase activity assays of FtsZ wild-type and mutant proteins indicate their functionality. Functionality of FtsZ was tested by comparing GTPase activities of full-length, wild-type proteins [BsFtsZ_1-382 (no tag)_ and BsFtsZ_1-382_] and individual mutants. With the exception of BsFtsZ_1-382 (no tag)_, depicted proteins were expressed with a C-terminal His-6 tag. All mutants, except for BsFtsZ_L272E_, as expected, retained GTPase activity mostly similar to the wild-type level. Accordingly, an E. coli FtsZ_L272E_ mutant was shown to bind nucleotides but was incapable of polymerizing. Also for the BsFtsZ-eGFP fusion, GTPase activity of FtsZ was slightly reduced, which may be due to a disturbance by the eGFP fusion partner. In all assays, 10 μM of protein was used and GTP turnover was measured after 10 min. The mean of three biological replicates is indicated; error bars show highest and lowest values of the replicates. Wild-type, untagged BsFtsZ [BsFtsZ_1-382 (no tag)_] was set to 100%. Download FIG S2, PDF file, 0.05 MB. Copyright © 2020 Silber et al. 2020 Silber et al. This content is distributed under the terms of the Creative Commons Attribution 4.0 International license.

10.1128/mBio.01006-20.3 FIG S3 ADEP derivatives differ in activating ClpP for the degradation of FtsZ. (A) Structure of the natural product ADEP1 and its synthetic congeners ADEP2, 4, and 7. ADEP1 (“factor A”) is a natural product of Streptomyces hawaiiensis NRRL 15010 (4). The synthetic congeners have been reported previously (1). Highlighted regions indicate where the synthetic congeners deviate from the natural product ADEP1. (B) SDS-PAGE analyses of *in vitro* ADEP-ClpP degradation assays using full-length BsFtsZ_1-382_ and BsClpP proteins in combination with different ADEP derivatives. Here, ADEP2 and ADEP4 were most effective in activating BsClpP. DMSO was used as a control (“-60 min”). ADEP2 was selected for all subsequent experiments. All experiments were performed at least in triplicate; representative images are depicted. Download FIG S3, PDF file, 0.7 MB. Copyright © 2020 Silber et al. 2020 Silber et al. This content is distributed under the terms of the Creative Commons Attribution 4.0 International license.

10.1128/mBio.01006-20.4 FIG S4 ADEP does not interfere with FtsZ GTPase activity. The target of ADEP is ClpP. To further exclude self-unfolding of FtsZ during incubation at 37°C as well as off-target effects of ADEP on FtsZ activity in our *in vitro* assays, we tested the functionality of FtsZ under these conditions via GTPase activity assays. Nonhydrolyzed GTP was read out by conversion to ATP to fuel a luciferase reaction. Here, GTPase activity of FtsZ remained unaffected upon a 60-min incubation at 37°C. Hence, FtsZ does not turn unstable or functionally inactive during our *in vitro* assays. Furthermore, low or high concentrations of ADEP (molar ratio of FtsZ:ADEP2 of 1:1 or 1:5, respectively) did not affect GTPase activity, indicating that there are no off-target effects of ADEP on FtsZ to be expected. Of note, in the *in vitro* degradation assays of this study, the ADEP concentration never surpassed the molar ratio for FtsZ:ADEP2 of 1:1.6. Download FIG S4, PDF file, 0.2 MB. Copyright © 2020 Silber et al. 2020 Silber et al. This content is distributed under the terms of the Creative Commons Attribution 4.0 International license.

### Hydrophobicity of the FtsZ N terminus is important for degradation.

**FIG 2:**
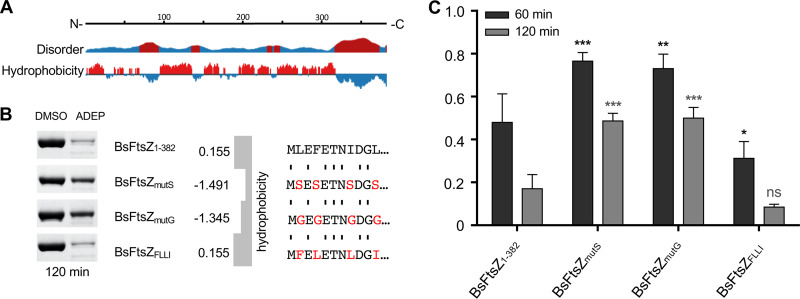
Hydrophobicity of the N terminus promotes FtsZ degradation. (A) Disorder prediction (top) and hydrophobicity prediction of FtsZ (bottom) were computed with JRONN software (66) (Protein Data Bank entry 2RHO; https://www.rcsb.org). Predicted disordered or hydrophobic regions are in red; ordered or hydrophilic regions are in blue. (B) ADEP-ClpP degradation assays of full-length, wild-type BsFtsZ_1-382_ as well as of BsFtsZ_mutS_ and BsFtsZ_mutG_ mutant proteins (left image), which carry mutations that change the hydrophobicity of their N termini (right image). In addition, a BsFtsZ_FLLI_ mutant protein was tested in which N-terminal hydrophobic amino acids were flipped, while overall hydrophobicity remained unchanged to wild-type FtsZ. The grand average of hydropathicity (GRAVY) of the mutated N termini was calculated according to the Kyte-Doolittle scale (67) using online software (https://web.expasy.org/protparam/). In these assays, low concentrations of ADEP and ClpP were used (1.5 μM ClpP; 1.5 μM ADEP). Samples were taken after a 120-min incubation; DMSO was used as a control. (C) Densitometry of protein bands from SDS-PAGE was performed to compare FtsZ protein amounts in ADEP-treated samples with the corresponding DMSO control reactions after 60 min (black bars) and 120 min (gray bars). The respective DMSO control was set to 1.0 (100%). For each FtsZ variant, the data were collected from three different degradation assays and SDS-PAGE analyses using a standard curve. The plotted data depict the corresponding mean values, with standard deviations indicated by error bars. Compared with BsFtsZ_1-382_, BsFtsZ_mutS_ and BsFtsZ_mutG_ were significantly less degraded by ADEP-ClpP after incubation for 60 or 120 min. In contrast, FtsZ_FLLI_ was similarly or slightly better degraded than BsFtsZ_1-382_. *, *P* < 0.05; **, *P* < 0.01; ***, *P* < 0.001.

The observation that ADEP-ClpP starts degradation at the N terminus of FtsZ was rather unexpected, raising the question of why the short N terminus should be preferred over the extended, flexible C terminus. To address this question, we analyzed the physicochemical properties of the N terminus of FtsZ *in silico*, and it emerged that it is overall hydrophobic in nature, in contrast to the C terminus of FtsZ (Fig. 2A). We thus hypothesized that hydrophobicity may play a role in determining FtsZ as a target for ADEP-ClpP at low antibiotic concentrations and constructed functional mutant proteins with varied hydrophobicity of the N terminus. And indeed, the less hydrophobic mutant proteins BsFtsZ_mutG_ and BsFtsZ_mutS_, for which all hydrophobic amino acids of the N terminus were replaced by either glycine or serine, respectively, were clearly less prone to degradation by ADEP-ClpP (Fig. 2B and C), while retaining functionality in control GTPase assays (Fig. S2). When we exchanged the positions of the individual hydrophobic amino acids in BsFtsZ_FLLI_ while keeping the overall hydrophobicity of this region unchanged, the mutant protein remained functional (Fig. S2) and was degraded similarly to full-length, wild-type BsFtsZ (Fig. 2B and C). This finding indicates that degradation of FtsZ does not depend on a certain amino acid sequence but that it is triggered by the hydrophobic nature of the N terminus.

### The N terminus of FtsZ does not act as a universal degradation tag for ADEP-ClpP.

**FIG 3:**
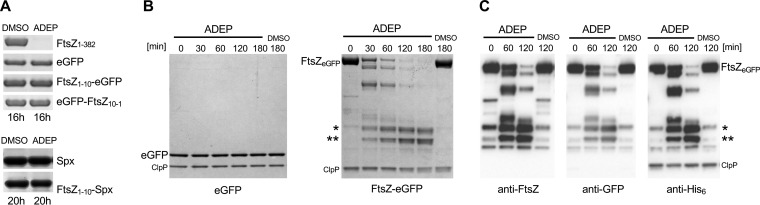
The N terminus of FtsZ is not sufficient to render proteins vulnerable to ADEP-ClpP. (A) *In vitro* ADEP-ClpP degradation assays of FtsZ_1-10_ fused to either eGFP or Spx. For the fusion of FtsZ_1-10_ to the C terminus of eGFP, the order of the N-terminal amino acids was inverted, which is indicated by FtsZ_10-1_. (B) *In vitro* ADEP-ClpP degradation assays using eGFP alone as well as a fusion of eGFP to the C terminus of full-length FtsZ (FtsZ-eGFP). Here, eGFP alone resisted degradation, while FtsZ-eGFP was rapidly degraded, generating two accumulating degradation fragments over time (indicated by asterisks). (C) Immunoblotting of FtsZ-eGFP samples from ADEP-ClpP degradation assays using anti-FtsZ, anti-eGFP, and anti-His_6_ antibodies. Of note, anti-FtsZ and anti-His_6_ antibodies can be used to prove the integrity of the eGFP protein, as these antibodies recognize the C terminus of FtsZ and eGFP, respectively. The two accumulating degradation fragments of FtsZ-eGFP (indicated by asterisks) could be detected with all three antibodies, indicating an intact eGFP protein. In all assays, high concentrations of ClpP and ADEP2 were used (2.5 μM ClpP, 6.25 μM ADEP2). Samples were taken at indicated time points. DMSO was used as a control. All experiments were performed at least in triplicate; representative SDS-PAGE (A, B) or Western blot (C) images are depicted.

We next wondered whether the hydrophobic N terminus of FtsZ may serve as a general degradation tag and would render proteins susceptible to ADEP-ClpP that otherwise resist degradation. To test this, we fused the N terminus of FtsZ (FtsZ_1-10_) to enhanced green fluorescent protein (eGFP), a common model substrate for joint ClpP/Clp-ATPase complexes. However, neither attaching FtsZ_1-10_ to the N terminus nor to the C terminus of eGFP resulted in its degradation by ADEP-ClpP (Fig. 3A). eGFP is known as a very stably folded protein that usually resists degradation by ClpP/Clp-ATPase complexes unless it is fused to a specific degron, which is recognized by the corresponding Clp-ATPase partner (28). Therefore, we also tested a fusion of FtsZ_1-10_ to the Clp substrate Spx, but again, we did not observe degradation even after prolonged incubation with ADEP-ClpP (Fig. 3A), demonstrating that FtsZ_1-10_ is not a degradation tag by itself that generally labels protein substrates for proteolysis by ADEP-ClpP. We then explored whether attaching the full-length FtsZ protein would trigger the degradation of eGFP by ADEP-ClpP. To this end, we constructed and purified a chimeric protein where eGFP is fused to the C terminus of full-length FtsZ, i.e., FtsZ-eGFP, which we then used in *in vitro* ADEP-ClpP degradation assays (Fig. 3B). As a control, we used eGFP without FtsZ fusion, which resisted degradation by ADEP-ClpP. In our assay, FtsZ-eGFP was rapidly degraded, with two distinct degradation fragments accumulating over time. We further characterized these two fragments by immunoblotting using anti-His_6_, anti-eGFP, and anti-FtsZ antibodies (Fig. 3C). Of note, the anti-FtsZ antibody used here recognizes the extreme C terminus of FtsZ, and the anti-His_6_ antibody binds to the C-terminal His_6_ tag of eGFP. Thus, anti-FtsZ and anti-His_6_ antibodies can be used to confirm the integrity of the N and C terminus of eGFP, respectively, in this construct. Here, immunoblots showed that both accumulating fragments comprised full-length eGFP, since corresponding signals could be detected via all three antibodies. Corroborating the degradation of FtsZ from the N terminus, here ADEP-ClpP degraded the FtsZ part of the fusion protein FtsZ-eGFP, but it did not succeed in degrading eGFP even when fused to FtsZ. Obviously, FtsZ possesses additional inherent characteristics that only in combination with the hydrophobic N terminus permit degradation by ADEP-ClpP.

### FtsZ cleavage extends into the folded N-terminal domain.

**FIG 4:**
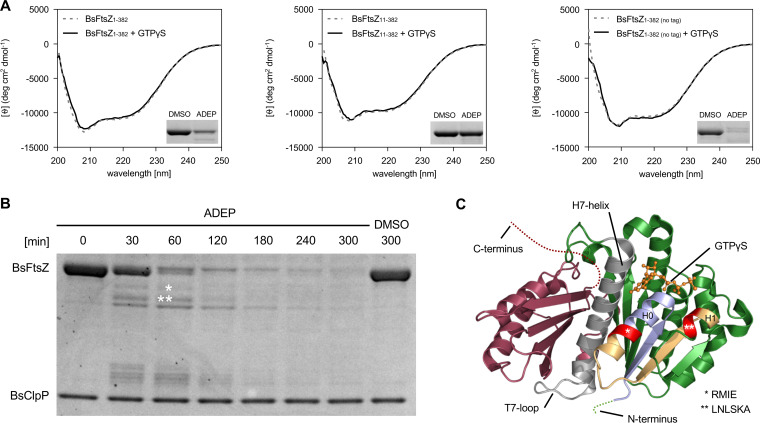
FtsZ folding and identification of early fragments of FtsZ degradation. (A) CD spectra of BsFtsZ_1-382_ and BsFtsZ_11-382_ (both C-terminally His_6_ tagged) as well as BsFtsZ_1-382 (no tag)_ in the absence (gray dashed line) or presence of GTPγS (black line). All samples show the characteristic ellipticity spectra for an alpha-helical fold with two minima at 208 nm and 222 nm, indicating a folded state of the FtsZ protein under these conditions. As a control, *in vitro* degradation of the corresponding BsFtsZ proteins (without GTPγS) was performed using the CD buffer conditions (i.e., activity buffer CD) and low concentrations of ADEP and ClpP (1.5 μM ClpP, 1.5 μM ADEP, and 4 μM FtsZ). Samples were taken after 60 min; DMSO was used as a control. Representative images of duplicates are depicted. (B) Time course of BsFtsZ_1-382_ degradation by ADEP-ClpP, indicating the generation of distinct degradation products. In these assays, low concentrations of ADEP and ClpP were used (1.5 μM ClpP, 1.5 μM ADEP, and 4 μM FtsZ). Samples were taken at indicated time points; DMSO was used as a control. Of note, when degradation reactions were allowed to continue, all depicted bands disappeared, indicating the capacity of ADEP-ClpP to fully digest FtsZ. The two largest of the emerging bands were excised (corresponding protein fragments are indicated by asterisks) and analyzed by Edman N-terminal protein sequencing. (C) Edman sequencing identified cleavage up to amino acid position 28 at the end of alpha-helix H0 (*RMIE) for the first fragment and up to position 49 of alpha helix H1 (**LNLSKA) for the second fragment (as indicated), which revealed that cleavage extends into the folded N-terminal domain of FtsZ. The crystal structure of GTPγS-bound BsFtsZ (Protein Data Bank entry 2RHO) highlights the first identified amino acid of both early fragments in red (the associated degradation product is indicated by asterisks) as well as the corresponding structural elements that are cleaved off by ADEP-ClpP (* in light blue, ** in light blue and yellow). GTPγS is indicated as the orange stick model.

FtsZ is efficiently degraded into smaller fragments, with a preferred degradation start at its N terminus (Fig. S1). However, one would expect that such degradation would require unfolding of the FtsZ protein in the absence of Clp-ATPases since the protein is presumably too large to access the proteolytic chamber in its folded form. One could speculate that the removal of the flexible N terminus (i.e., amino acids 1 to 10) might destabilize FtsZ such that it consequently unfolds on its own. However, our data demonstrate that the deletion of the flexible N-terminal portion leaves FtsZ folding intact. BsFtsZ_11-382_ is functionally active in GTP hydrolysis (Fig. S2), and the truncated mutant is more stable than BsFtsZ_1-382_ against degradation by ADEP-ClpP, making self-unfolding of the mutant protein highly unlikely. Furthermore, circular dichroism (CD) spectra of BsFtsZ_1-382_, BsFtsZ_11-382_ as well as untagged BsFtsZ in the absence or presence of the nonhydrolyzable GTP analogue guanosine-5′-*O*-(3-thiotriphosphate) (GTPγS) were superimposable. All spectra revealed the characteristic ellipticity of alpha-helical folds with two minima at 208 nm and 222 nm, indicating the presence of folded FtsZ proteins (without nucleotides as well as in the GTPγS-bound form) with similar average secondary structures under conditions that allow for the efficient degradation of full-length BsFtsZ (Fig. 4A). Therefore, we investigated whether ADEP-ClpP may be capable of destabilizing the fold of FtsZ during N-terminal attack to allow for further protein degradation.

To address this aspect of FtsZ proteolysis in more detail, we performed orienting analyses via label-free mass spectrometry (electrospray ionization-tandem mass spectrometry [ESI-MS]) of full-length and high-molecular-weight fragments of FtsZ, which indicated N-terminal truncations following degradation by ADEP-ClpP (see Fig. S5 in the supplemental material). To further corroborate this result, we next performed N-terminal protein sequencing by Edman degradation to identify early degradation fragments of wild-type FtsZ. Edman sequencing of the two largest degradation bands visible by SDS-PAGE (Fig. 4B) identified cleavage up to amino acid position 28 at the end of alpha-helix H0 (N-terminal amino acids determined by sequencing, RMIE) as well as up to position 49 of alpha helix H1 (N-terminal amino acids, LNLSKA). Thus, the data confirmed N-terminal degradation by ADEP-ClpP that extends into the folded region of the N-terminal domain of FtsZ (Fig. 4C).

10.1128/mBio.01006-20.5 FIG S5 ESI-MS of full-length and high-molecular-weight fragments of FtsZ indicate N-terminal truncations following degradation by ADEP-ClpP. (A) FtsZ was purified and incubated with ClpP in the presence of ADEP2 or DMSO (negative control) and subsequently separated by SDS-PAGE. Protein bands corresponding to FtsZ full-length protein in the control (1) and a fragment thereof appearing in the ADEP-treated sample (2) were excised from the gel, tryptically digested, and subjected to orienting LC-ESI-MS studies. Low concentrations of ADEP/ClpP (1.5 μM ClpP monomer; 1.5 μM ADEP) were used. (B) ESI-MS sequence coverages of FtsZ are highlighted in gray and show that the FtsZ fragment generated no N-terminal tryptic peptides compared with the full-length protein. Amino acid identification of the N-termini was then achieved using Edman protein sequencing (Fig. 4, main text). Download FIG S5, PDF file, 2.1 MB. Copyright © 2020 Silber et al. 2020 Silber et al. This content is distributed under the terms of the Creative Commons Attribution 4.0 International license.

### Nucleotide binding prevents N-terminal degradation by stabilizing FtsZ.

**FIG 5:**
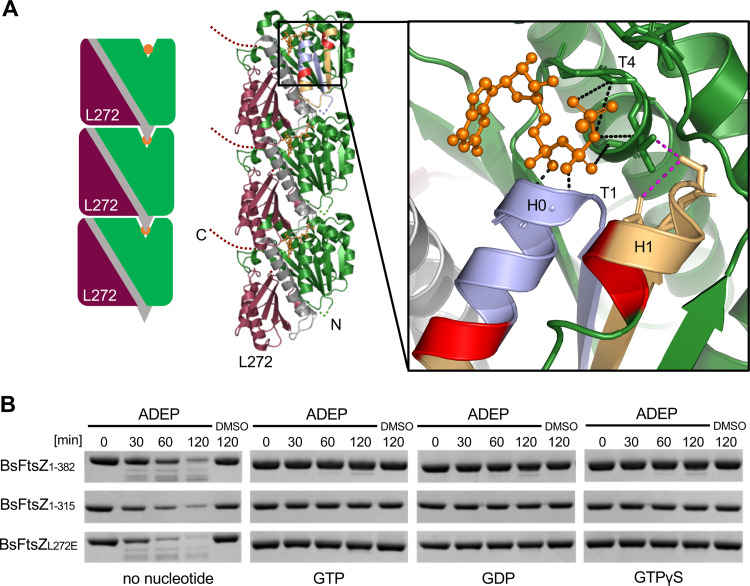
Nucleotide binding stabilizes FtsZ and inhibits degradation by ADEP-ClpP. (A) Head-to-tail association of individual FtsZ subunits result in single-stranded FtsZ protofilaments. The schematic shows the N-terminal domain (in green), the C-terminal domain (in currant red), and the central H7 helix with the T7 loop (in gray) as well as GTPγS (in orange). Also, the position of amino acid L272 inside the subunit interface is indicated. Note that in polymerized FtsZ, the short N terminus (N, green dotted line) is most probably largely buried in between two FtsZ monomers and may not be accessible to ADEP-ClpP. Zoom window shows established direct noncovalent contacts of GTPγS (in orange, Protein Data Bank entry 2RHO) with the T1 loop (Gly21-Gly22) and the T4 loop (Gly108-Gly110) (contacts in black) (37); the latter stabilizes helix H1 (contacts in magenta). The first amino acids of early FtsZ fragments, as identified by Edman sequencing (see Fig. 4), are indicated in red, cleaved off regions of the first and second degradation products in light blue and yellow, respectively. (B) ADEP-ClpP degradation assays of full-length BsFtsZ_1-382_ as well as of BsFtsZ_1-315_ and BsFtsZ_L272E_ in the absence or presence of either GTP, GDP, or GTPγS. In these assays, low concentrations of ADEP and ClpP were used (1.5 μM ClpP; 1.5 μM ADEP). Samples were taken at indicated time points, DMSO was used as a control. All results were confirmed by at least three independent experiments, and images of representative experiments are depicted.

During the course of cell division, FtsZ assembles into protofilaments to form the “Z-ring” at the prospective division site, providing the scaffold for the assembly of the bacterial cytokinetic machinery. The formation of single-stranded protofilaments depends on GTP binding to FtsZ, which results in a dynamic head-to-tail association of individual FtsZ subunits (27, 29–33). The short hydrophobic N terminus of FtsZ can be assumed to be largely buried inside the binding interface between two subunits of an FtsZ protofilament (Fig. 5A). Therefore, we wondered whether the addition of GTP would have an impact on FtsZ degradation by rendering the N terminus less accessible to ADEP-ClpP. To test this, we preincubated FtsZ with GTP prior to ADEP-ClpP degradation and, indeed, preincubated FtsZ (BsFtsZ_1-382_ and BsFtsZ_1-315_) clearly resisted degradation, in contrast to the control which showed substantial proteolytic degradation in the absence of GTP (Fig. 5B). Also, preincubation with GDP, which mostly generates dimers or shorter, curved filaments of FtsZ (34–36), clearly inhibited the degradation of FtsZ. As a simple explanation for the observed protection from degradation, it might be speculated that the N terminus of FtsZ is hidden inside the protofilament and may, thus, be inaccessible to ADEP-ClpP (Fig. 5A, middle image).

Intriguingly, the N-terminal cuts detected by Edman sequencing require helices H0 and H1 to be pulled out of FtsZ, and these helices are located in the vicinity of the nucleotide binding site (Fig. 5A, right). Noteworthy, bound nucleotides establish direct noncovalent contacts with the T1 loop (Gly21-Gly22) and T4 loop (Gly108-Gly110) (37), the latter being part of the tubulin signature motif (GGGTGTG) which stabilizes helix H1 (Fig. 5A, right). As we hypothesized that FtsZ has to undergo unfolding during N-terminal degradation by ADEP-ClpP, we wondered if the sole binding of a guanosine nucleotide, regardless of protofilament formation, may already stabilize helices H0 and H1, thereby preventing FtsZ unfolding and subsequent degradation. To approach this hypothesis, we explored the impact of GTPγS that does not support protofilament formation. FtsZ polymerization cannot be induced by GTPγS that, however, still binds to FtsZ (29, 38–41). And indeed, when we incubated FtsZ with GTPγS before the addition of ADEP-ClpP, thereby generating monomeric FtsZ bound to GTPγS, degradation was inhibited to the same extent as with GTP or GDP (Fig. 5B). To follow this route further, we employed a mutant FtsZ protein, BsFtsZ_L272E_, which is capable of nucleotide binding but is impaired in GTP hydrolysis (Fig. S2). Accordingly, an Escherichia coli FtsZ_L272E_ mutant had previously been described to exist only in the monomeric, nonpolymerized form, as L272 is located inside the biologically relevant intersubunit interface (42). In accordance with the results obtained for monomeric wild-type FtsZ bound to GTPγS, BsFtsZ_L272E_ resisted degradation by ADEP-ClpP when preincubated with either GTP, GDP, or GTPγS (Fig. 5B). Although FtsZ (without the addition of nucleotides) appears to be in a folded state with similar average secondary structures under conditions allowing FtsZ degradation *in vitro* (Fig. 4A), nucleotide binding alone is sufficient, without the need of protofilament formation, to further stabilize the overall fold of the N-terminal domain of FtsZ and to prevent degradation of FtsZ from the N terminus.

### The C terminus of FtsZ is increasingly degraded at higher concentrations of ADEP-ClpP.

**FIG 6:**
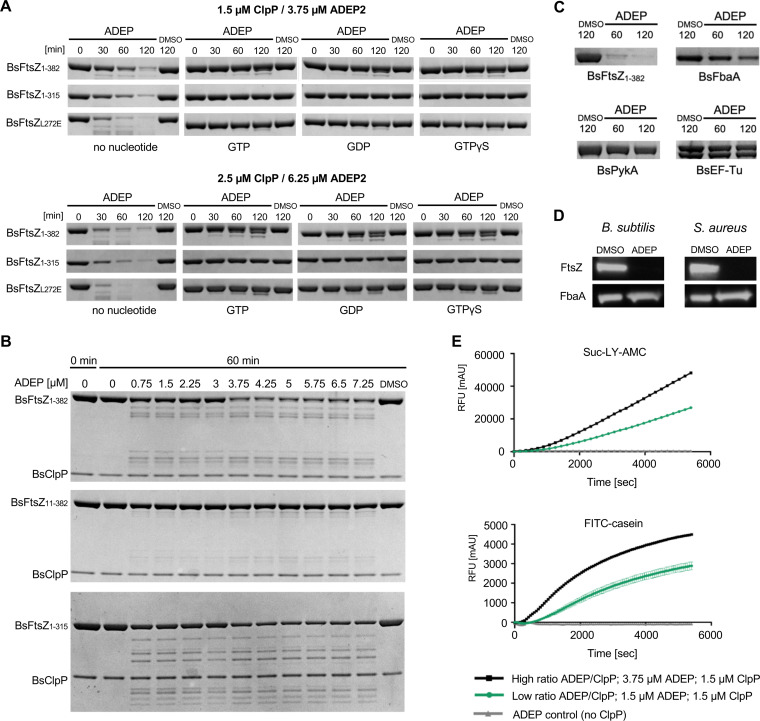
High concentrations of ADEP over ClpP lead to the degradation of the C terminus of FtsZ. (A) ADEP-ClpP degradation assays of BsFtsZ_1-382_, BsFtsZ_1-315_, or BsFtsZ_L272E_ in the absence or presence of guanosine nucleotides using increased concentrations of ADEP over ClpP (top, 1.5 μM ClpP and 3.75 μM ADEP2; bottom, 2.5 μM ClpP and 6.25 μM ADEP2). DMSO was used as a control. Results were confirmed by at least three independent experiments, and images of representative experiments are depicted. (B) Degradation of BsFtsZ_1-382_, BsFtsZ_11-382_, or BsFtsZ_1-315_ in the presence of 1.5 μM ClpP and increasing concentrations of ADEP2 (0 to 7.25 μM). Samples were taken after 60 min. DMSO was used as a control. Results were confirmed by at least three independent experiments, and images of representative experiments are depicted. (C) ADEP-ClpP degradation assays using BsFtsZ_1-382_, BsFbaA, BsEF-Tu, or BsPyk as substrates for ADEP-activated ClpP at a high concentration of ADEP over ClpP (2.5 μM ClpP; 6.25 μM ADEP). Samples were taken at indicated time points. DMSO was used as a control. Of note, phosphorylation of BsEF-Tu produces a doublet band resulting from partial phosphorylation of a multiply phosphorylated substrate (65). All experiments were performed at least in triplicate; representative images are depicted. (D) Immunoblotting of B. subtilis 168 or S. aureus NCTC8325 cells that were treated with high ADEP concentrations (3 μg/ml ADEP2 for B. subtilis 168 and 8 μg/ml ADEP2 for S. aureus NCTC 8325, i.e., 10× MIC), leading to growth arrest due to ceased biomass production. Here, immunoblots indicated the rapid degradation of FtsZ compared with the untreated control (DMSO). In contrast and despite the increased ADEP concentration, the abundance of FbaA remained unaltered in both species over time. Immunodetection of FtsZ or FbaA was performed using specific anti-FtsZ or anti-FbaA antibodies, respectively. Samples were taken after 120 min. (E) Effects of low and high concentrations of ADEP on peptidase activity using the fluorogenic peptide substrate Suc-LY-AMC (top) or on protease activity using the fluorogenic protein substrate FITC-casein (bottom), respectively. Quenched 7-amino-4-methylcoumarin (AMC) fluorescence is released upon peptide hydrolysis (top). Quenched FITC fluorescence is released upon proteolysis (bottom). Reaction rates per 1.5 μM BsClpP in relative fluorescence units (RFUs) during the linear phase of substrate degradation. DMSO was used in control reactions and was subtracted from corresponding ADEP values. The reaction in the absence of ClpP was used as an additional control. Assays were confirmed in at least three independent experiments. Error bars for Suc-LY-AMC and FITC-casein assays indicate standard deviations.

As detailed above, degradation of FtsZ by ADEP-ClpP preferentially starts from the N terminus, and low ADEP/ClpP concentrations (1.5 μM ClpP; 1.5 μM ADEP) were sufficient for efficient degradation of the entire protein into small fragments. FtsZ mutants lacking the short flexible N terminus remained largely intact when exposed to ADEP at these low concentrations. Nonetheless, in degradation assays using N-terminally truncated BsFtsZ_11-382_ (Fig. 1E) or nucleotide-bound FtsZ_1-382_ (Fig. 5B), a single faint band appeared slightly below the band of the full-length protein after prolonged incubation with ADEP-ClpP. The slow appearance of this band, its large size, and the fact that it was not readily degraded further oppose an origin from a fraction of residual unfolded or nucleotide-free FtsZ. Rather, these findings pointed toward a second target site within FtsZ to be degraded with considerably lower efficiency. We thus increased the concentration of ADEP and ClpP in our *in vitro* assay system (1.5 μM ClpP, 3.75 μM ADEP2 as well as 2.5 μM ClpP, 6.25 μM ADEP2; both correspond to 2.5 molar surplus of ADEP) and tested if we could also trigger degradation of nucleotide-bound FtsZ. And indeed, nucleotide-bound FtsZ was increasingly degraded at higher concentrations of ADEP/ClpP indicated by the appearance of more pronounced degradation fragments (Fig. 6A). Of note, degradation was also observed when using GTP, GDP, or GTPγS with FtsZ_L272E_. Since the N terminus is stabilized in nucleotide-bound FtsZ, we hypothesized that the C terminus might serve as a secondary target site at higher concentrations of ADEP/ClpP. To further explore this, we tested the degradation of nucleotide-bound BsFtsZ_1-315_ which lacks the flexible C terminus. In fact, the addition of nucleotides prevented the truncation of BsFtsZ_1-315_, and no degradation bands appeared even at higher concentrations of ADEP/ClpP (Fig. 6A; see Fig. S6A in the supplemental material). C-terminal attack was also confirmed *in vitro* by immunoblotting using a His_6_ tag fused to the C terminus of FtsZ (Fig. S6B). Likewise in whole cells, ADEP-ClpP truncated the C terminus of FtsZ when the N terminus was blocked with GFP, generating a stable degradation product only slightly smaller than FtsZ itself (see Fig. S7A in the supplemental material). In contrast, when GFP blocked the C terminus of FtsZ, FtsZ appeared fully degraded by ADEP-ClpP. Interestingly, we did not detect an accumulation of GFP fragments in the whole-cell situation that should have derived from an FtsZ-only degradation of the FtsZ-GFP fusion protein (Fig. S7B), as seen in our *in vitro* assays (Fig. 3C). Thus, we cannot exclude that FtsZ-GFP or fragments thereof may be fully degraded by ADEP-ClpP alone or with the help of other proteases in the bacterial cell, including degradation of GFP under these conditions.

10.1128/mBio.01006-20.6 FIG S6 The C terminus of FtsZ is an additional target site at high concentrations of ADEP/ClpP. (A) FtsZ_1-382_ or FtsZ_1-315_, both with attached C-terminal His_6_-tags, were preincubated with or without GTP and subsequently used in ADEP-ClpP degradation assays with a high concentration of ADEP/ClpP (2.5 μM ClpP; 6.25 μM ADEP2). SDS-PAGE images show two distinct degradation products for FtsZ_1-382_ after 120 min in the presence of ADEP-ClpP and GTP. Of note, no degradation bands were detected for FtsZ_1-315_ in the presence of ADEP-ClpP and GTP. DMSO was used as a control. (B) SDS-PAGE and corresponding Western blots using either anti-His_6_ or anti-FtsZ antibodies show that the degradation products of FtsZ_1-382_ lack the C-terminal His_6_-tag, proving C-terminal attack by ADEP-ClpP. Signals for anti-His_6_ and anti-FtsZ antibodies were intentionally overexposed (resulting in white regions within the protein band) to also allow the detection of weaker signals. Arrows mark the position of a C-terminal degradation product that could be detected by SDS-PAGE and with an anti-FtsZ antibody but not with an anti-His6 antibody. DMSO was used as a control. All experiments were performed at least in triplicate; representative images are depicted. Download FIG S6, PDF file, 1.0 MB. Copyright © 2020 Silber et al. 2020 Silber et al. This content is distributed under the terms of the Creative Commons Attribution 4.0 International license.

10.1128/mBio.01006-20.7 FIG S7 ADEP-ClpP targets both termini of FtsZ at increased ADEP concentrations in whole cells. Exponentially growing B. subtilis strains, which expressed both wild-type FtsZ as well as FtsZ mutant proteins attached to GFP (either fused to the N or to the C terminus of FtsZ) were treated for 60 minutes with either DMSO (negative control) or 0.5 μg/ml ADEP2, an antibiotic concentration that is well above the optimal filamentation concentration. Protein extracts were prepared and analyzed via immunoblotting using anti-FtsZ (A) and anti-GFP antibodies (B). In accordance with our *in vitro* data of nucleotide-bound FtsZ, blocking the hydrophobic N terminus of FtsZ with GFP only led to the truncation of the flexible C terminus of FtsZ, thereby generating a stable degradation product. On the contrary, when the C terminus of FtsZ was blocked by GFP and the N terminus remained accessible, FtsZ was fully degraded similar to wild-type FtsZ. Noteworthy, FtsZ-GFP runs sightly lower on SDS-PAGE than GFP-FtsZ which may be the result of the fusion of GFP to the long, flexible C terminus of FtsZ, probably providing more flexibility to the protein while running on SDS-PAGE than an N-terminal fusion. Detection of the DivIVA protein using an anti-DivIVA antibody (A) served as a loading control. Furthermore, we did not detect accumulating GFP fragments upon ADEP treatment of strain 2014 expressing the FtsZ-GFP fusion. Thus, we cannot exclude that GFP fragments of FtsZ-GFP may be further processed by either ADEP-ClpP or other proteases in the bacterial cell under these conditions. Images are representative of at least three independent experiments. Download FIG S7, PDF file, 0.5 MB. Copyright © 2020 Silber et al. 2020 Silber et al. This content is distributed under the terms of the Creative Commons Attribution 4.0 International license.

We then gradually increased the ADEP concentration in our *in vitro* degradation assay, while keeping the ClpP concentration constant at 1.5 μM, and analyzed the degradation of the proteins BsFtsZ_1-382_, BsFtsZ_11-382_, and BsFtsZ_1-315_ (Fig. 6B). Here, the degradation of BsFtsZ_1-382_ was clearly increased at more than 2-fold the molar concentration of ADEP (starting at 3.75 μM ADEP2) over the ClpP monomer concentration. In accordance with our data described above, the degradation of BsFtsZ_11-382_ was mostly prevented at lower ADEP/ClpP ratios but was triggered at higher ADEP concentrations, indicated by the appearance of pronounced degradation fragments, as a result of increased C-terminal degradation of FtsZ under these conditions. Also, N-terminal degradation of FtsZ was reproducibly accelerated at higher ADEP/ClpP ratios since BsFtsZ_1-315_ was also increasingly degraded. To further test an ADEP/ClpP concentration-dependent degradation of other proteins, we attempted the degradation of BsEF-Tu, BsPyk, and BsFbaA using high ADEP/ClpP concentrations in our *in vitro* assays (Fig. 6C). While BsEF-Tu and BsPyk still resisted degradation under these conditions, the amount of BsFbaA slightly decreased over time, suggesting its degradation by ADEP-ClpP. However, when we employed immunoblotting to test the abundance of FbaA in B. subtilis and S. aureus cells that had been treated with high ADEP concentrations (>10× MIC), the protein concentration of BsFbaA still remained unaltered under these conditions, in contrast to BsFtsZ (Fig. 6D). Thus, it cannot be excluded that either a portion of the FbaA protein batch used in our *in vitro* assay may not have been properly folded, which may lead to its degradation, or that FbaA is sufficiently replenished by protein expression or its fold may be stabilized in the bacterial cytoplasm, impeding its degradation.

Our data consistently indicate that the flexible C terminus of FtsZ is targeted at higher concentrations of ADEP/ClpP in addition to the N terminus. To explore the molecular rationale for this observation, we tested the peptidase and protease activity of ADEP-ClpP under the same assay conditions (low versus high concentration of ADEP/ClpP). By studying the degradation of the peptide substrate Suc-Leu-Tyr-7-amino-4-methylcoumarin (Suc-LY-AMC) (Fig. 6E, top graph) or the loosely folded protein model substrate fluorescein isothiocyanate-casein (FITC-casein) (Fig. 6E, bottom graph), both the peptidolytic and proteolytic degradation rate of ADEP-activated ClpP were notably increased when the concentration of ADEP was raised. However, it is a matter not only of degradation velocity but also of selection capability, as a further target structure (the C-terminal end in addition to the N terminus of FtsZ) is addressed under these conditions. Hence, our data indicate that the destructive quality of ClpP increases with rising concentrations of ADEP, i.e., the spectrum of efficiently degraded substrates by ADEP-ClpP is widened. In the treated cell, this probably reflects the degradation of additional target structures, of which the C terminus of FtsZ is only but one example. Our results on FtsZ thus show that the fate of a protein, when targeted by ADEP-ClpP, depends not only on the capabilities of the deregulated protease but also on protein-intrinsic fragile or stabilizing substructures as well as physiochemical properties.

## DISCUSSION

ADEP antibiotics have a dual mechanism of antibacterial action that is based on the multilayered deregulation of bacterial protease Clp; by binding to ClpP, ADEP inhibits all Clp functions in the bacterial cell (13, 14), and at the same time, it activates ClpP for the proteolysis of nonnative substrates in the absence of regulatory Clp-ATPases (1, 13, 17, 22). Depending on the bacterial species, ADEP uses either one or the other mechanisms for bacterial killing (1, 14). With regard to ClpP activation for the Clp-ATPase-independent degradation of nonnative substrates, we previously observed the following two concentration-dependent phenotypes in B. subtilis: (i) at ADEP concentrations several times the MIC, biomass increase ceased early (22, 23) and treated cells remained small, suggesting a depletion of essential proteins in various processes of the bacterial metabolism; and (ii) in contrast, at ADEP concentrations close to the MIC, treated cells were characterized by a phenotype of extensive filamentation, clearly marking an inhibition of cell division as the major antibiotic effect at these antibiotic concentrations. This filamentation phenotype is due to the untimely degradation of FtsZ (22), the pacemaker protein of cell division in most bacteria (30, 31). In fact, our new data show that the FtsZ protein seems especially prone to degradation compared with other proteins, such as FbaA, EF-Tu, or Pyk, although FtsZ is most probably not the only cellular protein substrate of ADEP-ClpP, and nascent polypeptides at the ribosome are ADEP-ClpP targets as well (1, 13). In this context, it is further noteworthy that ADEP-activated BsClpP is even capable of degrading FtsZ from different species, including Mycobacterium tuberculosis (14). However, the reason for such a preferential degradation of FtsZ at low inhibitory ADEP concentrations has remained elusive so far.

In the current report, we now provide insight into the mechanism of FtsZ degradation by ADEP-ClpP which emerged to occur stepwise at different structural sites of FtsZ, depending on the applied concentration of ADEP and ClpP. At low concentrations of ADEP/ClpP, our results show that degradation by ADEP-ClpP preferably starts at the short N terminus of FtsZ. This finding was rather unexpected because the N-terminal region of FtsZ, which extends beyond the globular protein structure, is too short to reach the secluded catalytic sites of the ADEP-activated ClpP tetradecamer without the need of unfolding FtsZ. Assuming that the accessible part of the FtsZ N terminus is an extended peptide chain without secondary structure, its calculated length according to the Pauling model would be 36 Å (10 amino acids, 3.6 Å each for an extended peptide chain) (43, 44). However, a polypeptide chain would need to cover a distance of at least 40 Å from the entrance pore of the ADEP-ClpP tetradecamer (Glu53) to the active site serine (S97) of the catalytic triads (19). In contrast, the extended, flexible C terminus of FtsZ with a theoretical length of more than 200 Å would be long enough by far to easily diffuse through the opened pores of a ClpP tetradecamer to reach the catalytic triad and be degraded. Although the entrance pore diameter changes from ∼1.8 nm in apo-BsClpP to ∼2.7 nm in ADEP-bound BsClpP (19), it is still not wide enough to allow for entry of the N-terminal domain of FtsZ in its folded state (diameter, ∼4 nm) into the degradation chamber, which implies that the N terminus of FtsZ needs to unfold during attack by ADEP-ClpP. A mechanism of protein unfolding is further supported by our observation that nucleotide binding to FtsZ prevents degradation at the N terminus, most probably by stabilizing the overall fold of the N-terminal domain of FtsZ. To our best knowledge, ClpP has not been described before to lead to protein unfolding in the absence of an associated Clp-ATPase, which usually unfolds Clp target proteins in an energy-dependent manner and upon recognition of a specific degron, such as SsrA (28). In Escherichia coli, for example, ClpX identifies FtsZ as a Clp target via two recognition signals located near the C terminus of FtsZ (45, 46). In this context, we have previously attempted the degradation of native Clp protease substrates using ADEP-ClpP, including MecA, McsB, and ComK from B. subtilis or DnaK, TigA, or GroEL from E. coli, as well as SsrA-tagged GFP, which all resisted degradation by ADEP-ClpP in our *in vitro* assays (22). On the other hand, FtsZ appears not to be degraded by ClpXP in B. subtilis (47, 48), indicating that the recognition of substrates by the Clp protease fundamentally differs from the substrate preference of ADEP-ClpP.

FtsZ has not principally been regarded as intrinsically unstable or loosely folded (25, 26, 30, 31). However, as FtsZ is a preferential target of ADEP-ClpP, which implies special inherent characteristics of the FtsZ protein, it may well be that the stability of the N-terminal domain of FtsZ (when devoid of nucleotides) is lower than that of other cytoplasmic proteins (49). Several studies reported on the presence of an unfolding intermediate of FtsZ and suggested that intradomain stabilization is important for overall protein stability (49–52). Since the N- and C-terminal domains of FtsZ represent independent folding domains (24), one could envision that partial unfolding of FtsZ, where the N-terminal half becomes unfolded and unbound from the C-terminal half of the polymerization domain, would allow for the exposure of a region smaller than the 2.7-nm diameter of the ADEP-bound BsClpP pore. Very recently, it has been reported that under certain *in vitro* conditions S. aureus FtsZ would occur in an unfolded state and requires nucleotide binding for proper folding (53). Although FtsZ was not intrinsically unfolded in our *in vitro* conditions, as shown by our CD data that indicate a normal FtsZ secondary structure fold with no difference in the absence or presence of nucleotides, this finding is very intriguing regarding the general stability of the FtsZ protein. Our data on the preferred degradation of FtsZ by ADEP-ClpP further support the notion of a rather fragile fold of the FtsZ protein (under *in vitro* conditions as well as in the living cell) compared with other tested cellular proteins, including natural Clp substrates or eGFP. We therefore suggest that upon direct binding, ADEP-ClpP exploits such intrinsic conformational flexibility of the folded FtsZ protein (or at least of its N-terminal domain), leading to protein unfolding and subsequent digestion. Such intrinsic flexibility of a protein’s fold may be a prerequisite for protein substrates of ADEP-ClpP, a degradative machine not supported by protein unfolding via energy-dependent Clp-ATPases.

**FIG 7:**
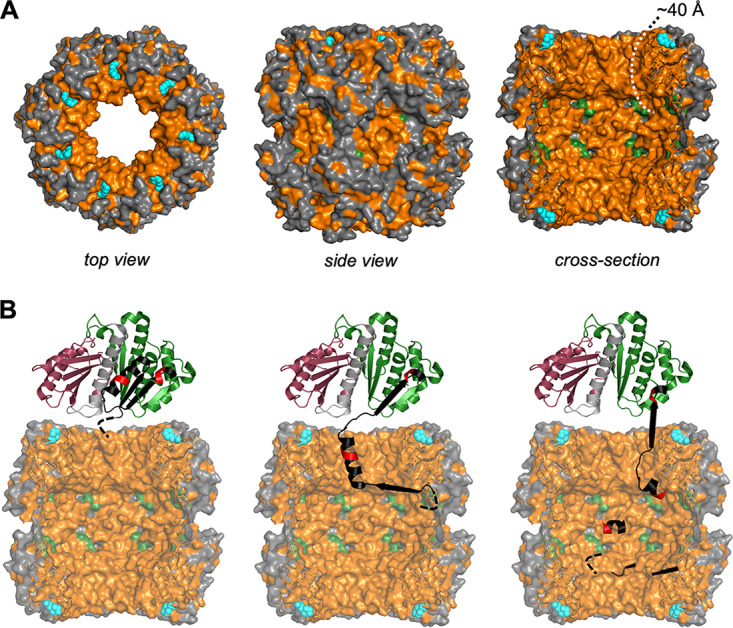
Proposed model for the N-terminal degradation of FtsZ by ADEP-ClpP. (A) Crystal structure of a B. subtilis ClpP tetradecamer (BsClpP; Protein Data Bank entry 3KTK; in gray with overall hydrophobic regions in orange) with ADEP2 bound to the hydrophobic pockets (cyan). Catalytic triads are highlighted in green. The cross-section of a BsClpP tetradecamer (right) models the inside of the proteolytic chamber, illustrating the overall hydrophobic nature of the ClpP entrance pore (54) and the inner surface of the degradation chamber (55). Noteworthy, in the available BsClpP crystal structure, the outermost N-terminal amino acids lining the entrance pore are not visible due to inherent flexibility. Thus, the actual situation regarding the hydrophobic surface interacting with an unfolded FtsZ N terminus or any unfolded protein will slightly deviate from what is shown here. To reach the catalytic triads within the proteolytic chamber, a peptide chain has to span a length of approximately 40 Å. (B) Hydrophobic interactions appear to be important for the attack of ADEP-ClpP on the N-terminal region of FtsZ since the hydrophobicity of the FtsZ N terminus supports its degradation. We propose that the hydrophobic N terminus of FtsZ (black dashed line) engages with ClpP via manifold hydrophobic interactions that occur within the entrance pore of ClpP and beyond within the degradation chamber. A sum of interactions that is, in total, stronger than the binding forces within the FtsZ N-terminal domain itself, supported by a nonstatic nature of the ClpP pore surface, might trigger an unfolding process that allows the N terminus of FtsZ to extend further and yet further into the catalytic chamber. Although ClpP alone might not “pull” by force, as the Clp-ATPases can do during their ATP-fueled power stroke, it might still be perfectly capable of “holding tight” long enough to destabilize the secondary structure within the potentially rather flexible N-terminal domain of nucleotide-free FtsZ (in black and green). Nucleotide binding, however, prevents FtsZ unfolding by stabilizing the N-terminal domain and thus inhibits degradation of the N terminus.

Our data show that hydrophobic interactions play a pivotal role in the degradation of the N-terminal region of FtsZ by ADEP-ClpP, which may provide an explanation for the observed phenomenon. Hydrophobic residues also cluster around the rim of the ClpP entrance pore (54), and the inner surface of the degradation chamber is largely hydrophobic as well (55). Since the hydrophobic effect is considered the major driving force for the folding of proteins, strong enough to trigger supramolecular assemblies (56), establishing new hydrophobic contacts between FtsZ and ADEP-ClpP might weaken intrinsic contacts within FtsZ itself and lead to its unfolding. We therefore propose that the N terminus of FtsZ establishes hydrophobic contacts with the entrance pore of ClpP and beyond within the degradation chamber and that the sum of such interactions might be sufficient to allow the N terminus of FtsZ to partially unfold and stretch further into the degradation chamber of ClpP, leading to a destabilization of the N-terminal protein fold of nucleotide-free FtsZ (Fig. 7).

At higher concentrations of ADEP/ClpP, our data show that the spectrum of effectively degraded substrates by ADEP-ClpP is broadened, as we observed the additional degradation of the C terminus of FtsZ when using excess ADEP over ClpP, a substructure which mostly resisted degradation at lower concentrations of ADEP/ClpP. The molecular reason for such a broadening of the ADEP-ClpP substrate spectrum remains currently unclear and deserves further study. However, although equimolar concentrations of ADEP and ClpP were used for the condition of “low concentration of ADEP/ClpP” in our assays, it is feasible that the ClpP tetradecamer may not be saturated with ADEP molecules under these conditions. In all available crystal structures of ADEP-ClpP, all hydrophobic pockets of ClpP were fully occupied (2, 19, 20, 57, 58), the entrance pores were widened to the extent described above, and all catalytic sites were captured in the active conformation. However, crystal structures present a static endpoint generated at very high protein and compound concentrations. In a dynamic constellation, lower versus higher concentrations of ADEP may well influence ClpP occupancy, pore dynamics, and active site activity, which may lead to a preference for certain targets, such as the N terminus of FtsZ, over others. In our study, we observed a two-step activation mechanism of ClpP regarding FtsZ degradation, where degradation efficiency visibly increased at more than 2-fold the molar excess of ADEP over ClpP (Fig. 6B), that may therefore reflect unsaturated and saturated states of the ClpP tetradecamer with respect to ADEP binding.

FtsZ assembles into protofilaments to eventually form the Z-ring at midcell and initiate cell division. FtsZ protofilament formation is characterized by a continuous binding and consumption of GTP, which is then released as GDP from FtsZ to allow the binding of a new GTP molecule. This constant exchange of nucleotides is accompanied by the dynamic exchange of FtsZ subunits between the Z-ring and the cytoplasmic pool (27, 29–33). Importantly, a certain critical concentration of FtsZ is required for protofilament formation (29, 59). Our data show that nucleotide-bound FtsZ resists proteolytic attack at lower ADEP concentrations. In the context of bacterial cell division, it may therefore be hypothesized that ADEP-ClpP will predominantly deplete the cytoplasmic pool of apo-FtsZ when low inhibitory ADEP concentrations are used. This may consequently lead to a reduction of the cytoplasmic FtsZ level below the critical concentration required for filamentation, which will inevitably result in an inhibition of cell division and eventually cell death. At higher ADEP concentrations, we showed that nucleotide binding does not prevent FtsZ degradation by ADEP-ClpP since the C terminus becomes an additional target independent of the presence of bound nucleotides. Here, the grappling hook peptide (GHP) (60) located at the extreme C terminus of FtsZ is degraded, which represents the interaction interface of FtsZ with other divisome proteins. Hence, C-terminal degradation will further contribute to a depletion of intact FtsZ available for Z-ring formation, thus accelerating the detrimental effects on cell division.

In conclusion, our data identify the hydrophobic N terminus of FtsZ as a preferred target at low levels of ADEP/ClpP, although it cannot be excluded that further proteins are also degraded at filamentation concentrations but show a less prominent phenotype. A stepwise degradation of distinct target structures by ADEP-ClpP as indicated here provides a rationale for the series of events that lead to the strikingly different phenotypes at low versus high ADEP concentrations (prolonged filamentation versus rapid cessation of biomass production, respectively) (23). This suicide-like mechanism of protease activation with its broad destructive capacity is distinct from all clinically applied antibiotics and makes ADEPs a promising starting point for the development of novel strategies for antibacterial attack.

## MATERIALS AND METHODS

### Bacterial strains and growth conditions.

Bacterial strains and plasmids used in this study are listed in Table S1 in the supplemental material. Bacteria were grown in lysogeny broth (LB) at 37°C, which was supplemented with appropriate antibiotics or inducing compounds when required. ADEP2 was added to early-exponential-phase B. subtilis or S. aureus cultures unless otherwise stated. For low (2× to 3× MIC) or high (>10× MIC) ADEP2 concentrations, 0.25 μg/ml or 3 μg/ml ADEP2 was used for B. subtilis and 1 μg/ml or 8 μg/ml ADEP2 for S. aureus, respectively. Of note, the ADEP per ClpP (ADEP/ClpP) molar ratios occurring in bacterial cells when exposed to the low and high ADEP concentrations in our whole-cell assays may, of course, differ from the ADEP/ClpP ratios adjusted in our *in vitro* assays. The actual ADEP concentrations in the two distinct settings were chosen for the following reasons: *in vitro*, the low and high concentrations were selected to clearly differentiate between the N-terminally focused degradation and the combined N-/C-terminal attack. In the whole-cell context, the low concentration leads to a strong filamentation phenotype in B. subtilis (respective swelling in S. aureus) and thus a phenotype dominated by FtsZ degradation, whereas under the high concentration, biomass increase and metabolism in general are severely inhibited, demonstrating degradation of a wider range of substrates. MICs were determine according to the guidelines of the Clinical and Laboratory Standards Institute (CLSI) except for using LB.

10.1128/mBio.01006-20.9 TABLE S1 Bacterial strains and plasmids. Download Table S1, PDF file, 0.1 MB. Copyright © 2020 Silber et al. 2020 Silber et al. This content is distributed under the terms of the Creative Commons Attribution 4.0 International license.

### Cloning experiments.

Primers that were used for cloning experiments in this study are listed in Table S2 in the supplemental material. Polymerase, T4 ligase, and antarctic phosphatase as well as restriction enzymes that were used for cloning experiments were obtained from New England BioLabs (NEB). Genes were amplified from genomic DNA of B. subtilis 168 (*clpP*, *ftsZ*, *fbaA*, *pyk*, and *tufA*) or S. aureus NCTC 8325 (*clpP* and *ftsZ*) (Table S1 and S2). The sequence of *egfp* was amplified using the plasmid pDest007-eGFP-(Ec)-ssrA (17). For ligation, a vector:insert ratio of 1:5 was employed using 70 ng of the double digested and dephosphorylated vector. PCR products were purified and digested with respective enzymes and ligated into expression vector pET22b or pET11a as indicated. For attaching purification tags or the N terminus of FtsZ, corresponding DNA fragments were amplified from template plasmids (indicated in Table S2) using either 5′-phosphorylated oligonucleotides or oligonucleotides with the same restriction site. The plasmids pET11a-bsPyk and pETftsZbs-egfp_H6_ were cloned using the Gibson assembly master mix (NEB). For plasmid pETftsZbs-egfp_H6_, a linker comprising 24 bp was inserted between the sequence of *ftsZ* and *egfp* to support correct protein folding and to allow sufficient flexibility of the protein fusion (the linker region is highlighted in gray in Table S2). Plasmid pETftsZbs_L272E_ was generated using the QuikChange II site-directed mutagenesis kit (Agilent Technologies). Primers were constructed as described according to the manufacturer’s manual.

10.1128/mBio.01006-20.10 TABLE S2 Primers used in this study. Download Table S2, PDF file, 0.04 MB. Copyright © 2020 Silber et al. 2020 Silber et al. This content is distributed under the terms of the Creative Commons Attribution 4.0 International license.

### Protein purification of ClpP and FtsZ.

Native FtsZ of B. subtilis 168 was expressed in E. coli strain W3110 (pBS58) (pCXZ), as described earlier (22, 61). His_6_-tagged FtsZ and ClpP proteins (originating from B. subtilis 168 or S. aureus NCTC8325) were expressed in E. coli BL21(DE3) harboring the respective expression plasmid (Table S1) and were purified as previously described (62). The quality and quantity of purified proteins were verified by SDS-PAGE, Bradford assay (using bovine serum albumin as the control), and spectrophotometry (Nanodrop Technologies). All tested FtsZ mutant proteins in this study were catalytically active, as shown by GTPase activity assays (GTPase-Glo assay; Promega) (Fig. S2), and the use of purification tags had no effect on degradation preference (see Fig. S8 in the supplemental material).

10.1128/mBio.01006-20.8 FIG S8 Streptavidin or His_6_ purification tags do not alter the degradation preference. ADEP-ClpP degradation assays (high ADEP/ClpP concentration: 2.5 μM ClpP; 6.25 μM ADEP) using full-length BsFtsZ_1-382_ proteins with a His_6_ tag fused to the N terminus and a Strep-tag attached to the C terminus of FtsZ (A; His_6_-FtsZ-Strep) or vice versa (B; Strep-FtsZ-His_6_). DMSO was used as a control. Immunoblots were generated using anti-Strep- or anti-His_6_-specific antibodies as indicated. The ladder of bands that appeared as the typical degradation pattern for FtsZ can only be detected with tags attached to the C terminus, confirming preferential targeting of the N terminus by ADEP-ClpP. Furthermore, the data show that these protein tags do not notably alter the degradation preference of ADEP-ClpP. Signals for anti-His_6_ and anti-Strep antibodies are intentionally overexposed (resulting in white regions within the protein band) to also allow the detection of weaker signals in the area of emerging degradation products. All experiments were performed at least in triplicate; representative images are depicted. Download FIG S8, PDF file, 0.3 MB. Copyright © 2020 Silber et al. 2020 Silber et al. This content is distributed under the terms of the Creative Commons Attribution 4.0 International license.

### GTPase activity assays.

The functionality of wild-type and mutant FtsZ proteins was analyzed using the GTPase-Glo assay (Promega) according to the manufacturer’s instructions (Fig. S2). The assay measures the amount of residual GTP after a GTPase reaction by converting remaining GTP into ATP that is then detected using a luciferin/luciferase reaction. Different FtsZ concentrations were incubated in a white 384-well microtiter plate (Brand) for 1 h at 25°C in the presence of 5 μM GTP and 1 mM DTT in GTPase/GAP buffer (volume, 10 μl). An equal volume of GTPase/Glo buffer was then added, and the reaction mixture was incubated for 30 min at 25°C while shaking. For the luciferin/luciferase reaction, 20 μl of the detection reagent was added to each well, and the luminescence was measured in a microplate reader (Tecan Infinite M200) after 10 min.

### Circular dichroism.

FtsZ proteins were used at a concentration of 3.5 μM in activity buffer CD (50 mM Tris/HCl [pH 8], 25 mM MgCl_2_, and 100 mM KCl) in the absence or presence of 40 μM GTPγS. To allow nucleotide binding by FtsZ, samples were preincubated for 10 min at room temperature before the start of the measurement. CD spectra were recorded from 200 to 250 nm (1-nm bandwidth, 20-nm min^−1^ scanning speed) with a Jasco J-720 spectropolarimeter using a 1-mm cuvette. The spectra were recorded 10 times for each protein sample, averaged, and corrected for the buffer system (with or without GTPγS, respectively). Spectra were only shown in the range of 200 nm to 250 nm since the activity buffer CD contains ions that strongly absorb at lower wavelengths.

### *In vitro* nucleotide binding and degradation assays.

For *in vitro* guanosine nucleotide binding, FtsZ proteins (4 μM) were incubated in activity buffer BS (50 mM Tris/HCl [pH 8], 25 mM MgCl_2_, and 100 mM KCl, 2 mM DTT) for B. subtilis proteins or activity buffer SA (20 mM HEPES [pH 7], 100 mM NaCl) for S. aureus proteins. Desired guanosine nucleotides were added to the reaction mixture to a final concentration of 1 mM according to a method described previously (63). For *in vitro* degradation, target proteins (4 μM BsPyk, BsEF-Tu, BsFbaA, eGFP, BsFtsZ-eGFP, and nucleotide-free or nucleotide-bound FtsZ) were incubated at 37°C in activity buffer BS (for B. subtilis proteins), activity buffer SA (for S. aureus proteins), or activity buffer CD (for CD spectroscopy control assays) in the presence of either low (1.5 μM ClpP; 1.5 μM ADEP) or high (1.5 μM ClpP; 3.75 μM ADEP, or 2.5 μM ClpP; 6.25 μM ADEP) concentrations of ADEP2 and ClpP monomer as indicated. Samples were taken at indicated time points and were analyzed via SDS-PAGE and immunodetection techniques (64). Where appropriate, protein amounts were calculated from SDS-PAGE band intensities measured via densitometry using Image Lab software (Bio-Rad). For quantitative/comparative analyses, a standard curve was generated representing 1 μg, 2 μg, 3 μg, 4 μg, and 5 μg of the control protein BsFtsZ_1-382_, which was loaded on the same SDS-PAGE as the protein samples derived from degradation experiments. Remaining FtsZ protein amounts in the ADEP-treated samples were then compared and normalized to the respective DMSO control reaction which was set to 100%. The data were plotted using mean values collected from three different degradation experiments and SDS-PAGE analyses for each FtsZ variant, with corresponding standard deviations indicated by error bars.

### Suc-LY-AMC and FITC-casein degradation assays.

For the degradation of the fluorescent peptide substrate Suc-LY-AMC, concentrations of ADEP2 and ClpP (monomer) were used as indicated. Peptidase assays were performed in activity buffer BS using 1.5 μM of purified ClpP protein and 400 μM of Suc-LY-AMC (dissolved in DMSO) in 100-μl reaction volumes. For the degradation of the fluorogenic protein fluorescein isothiocyanate-casein (FITC-casein; Sigma), concentrations of ADEP2 and ClpP (monomer) were used as indicated. Protease assays were performed in activity buffer BS using 1.5 μM of purified ClpP protein and 20 μM FITC-casein in 100-μl reaction volumes. In both peptidase and protease assays, an equal volume of DMSO compared to ADEP was used as a control. Corresponding DMSO samples were used as the baseline control and were subtracted from ADEP values. Hydrolysis of the peptide or protein substrates (indicated by fluorescence emission) was monitored in black, flat-bottom 96-well microplates (Sarstedt) at 37°C via measuring the release of either AMC or FITC, respectively, in a spectrofluorometer (Tecan Infinite M200) at an excitation wavelength (λex) of 380 nm and an emission wavelength (λem) of 460 nm (for Suc-LY-AMC) or at an λex of 490 nm and an λem of 525 nm (for FITC-casein).

### Immunoblotting of FtsZ, GFP, and FbaA.

To analyze the degradation of GFP-fused FtsZ in whole cells, B. subtilis strains 2014 and 2020 were cultivated in LB medium containing 1 mM isopropyl-β-d-thiogalactopyranoside (IPTG) and 0.5% xylose. Degradation of FtsZ and FbaA was further investigated using B. subtilis strain 168 as well as S. aureus strain NCTC 8325. Overnight cultures of all strains were diluted 1:200 in fresh LB (with or without appropriate inducers) and grown to an optical density at 600 nm (OD_600_) of 0.1 to 0.25 at 37°C while shaking. Then, cultures were split, adding either ADEP2 or DMSO as a negative control. For B. subtilis strains 2014 and 2020, 0.5 μg/ml ADEP2 was used. For B. subtilis 168 and S. aureus NCTC 8325, 0.25 μg/ml or 3 μg/ml and 1 μg/ml or 8 μg/ml ADEP2 were used for low or high concentrations, respectively. Cultures were further shaken at 37°C for 60 or 120 minutes (as indicated). Cells were then harvested, suspended in 0.5 to 1 ml lysis buffer (50 mM NaH_2_PO_4_ and 300 mM NaCl [pH 8], with added EDTA-free mini protease inhibitor; Roche), and lysed via cell disruption in a PreCellys homogenizer (6,800 rpm, 3 times for 20 s) using a mixture of 0.1-μm and 0.5-μm glass beads in 0.5-ml PreCellys tubes. The protein content of the cell lysates was measured by Nanodrop spectrophotometry and adjusted accordingly. Immunoblotting of whole-cell extracts or purified proteins was performed by standard techniques (64) using anti-FtsZ, anti-GFP, anti-His_6_, anti-FbaA, or anti-Div4A antibodies as indicated. A Coomassie blue-stained SDS-PAGE was additionally used to control the applied protein amount.

### Protein identification by mass spectrometry.

For orienting analyses, a partially truncated FtsZ protein was analyzed by label-free mass spectrometry (MS) using a Synapt G2-S HDMS time of flight (TOF) mass spectrometer equipped with an ESI NanoLockSpray source coupled online to a nanoAcquity ultraperformance liquid chromatography (UPLC) system and operated with MassLynx software (version V4.1 SCN932; Waters). Proteins were excised from the gel and destained twice in washing solution (20 mM ammonium bicarbonate, 30% acetonitrile). For reduction of disulfide bonds, gel pieces were incubated with 10 mM DTT in washing solution at 60°C for 45 min. Alkylation of cysteines was then performed with 50 mM iodoacetamide (IAA) in washing solution for 25 min at room temperature in the dark. Gel pieces were washed again twice with washing solution for 5 min at room temperature and dried using a vacuum centrifuge at 40°C prior to tryptic digestion with 6.25 ng/μl trypsin (Promega) in washing solution for 16 h at 37°C. Tryptic peptides were eluted using 20 μl of 0.1% trifluoroacetic acid and an ultrasonic bath for 15 min. Tryptic peptides were purified using a nanoAcquity UPLC trap symmetry C_18_ column (pore size, 100 Å; particle size, 5 μm; length, 20 mm; Waters) with a flow of 10 μl/min in 0.5% buffer B for 4 min. Tryptic peptides were then eluted from a nanoAcquity UPLC CSH130 C_18_ column (pore size, 130 Å; particle size, 1.7 μm; length, 100 mm; Waters) with a flow of 0.35 μl/min at 40°C using the following gradient: initial, 0.5% eluent B; 22 min, 60% eluent B; 24 min, 90% eluent B; 26 min, 99% eluent B; 27 min, 0.5% eluent B; and 30 min, 0.5% eluent B. Continuous MS^E^ spectra were recorded in a mass range from 50 to 1800 *m/z* and with a scan time of 1 s in positive resolution mode with the following settings: capillary voltage, 2.1 kV; cone voltage, 30 V; source temperature, 100°C; cone gas flow, 50 liters/h; desolvation gas flow, 550 liters/h; and desolvation temperature, 150°C. Collision energy was ramped from 14 to 45 eV. Serving as mass reference, leucine-encephalin was injected with a capillary voltage of 3 kV every 60 s. The mass spectra were processed with ProteinLynx Global Server (version 2.5.2; Waters). Processing parameters were adjusted as follows: chromatographic peak width, automatic; MS TOF resolution, automatic; lock mass for charge 1, 556.2771 Da/e; lock mass window, 0.25 Da; low energy threshold, 2,000 counts; elevated energy threshold, 500 counts; and intensity threshold, 10,000 counts. For protein identification, a data bank containing 4,156 proteins of E. coli BL21 (Uniprot reference sequence UP000002032, added manually; B. subtilis FtsZ and ClpP, trypsin, and keratin) was used. The following settings were used: peptide tolerance, automatic; fragment tolerance, automatic; min fragment ion matches per peptide, 5; min fragment ion matches per protein, 5; min peptide matches per protein, 1; maximum protein mass, 400,000; primary digest reagent, trypsin; secondary digest reagent, none; missed cleavages, 1; fixed modifications, carbamidomethyl C; variable modifications, deamidation N, deamidation Q, oxidation M; and false-positive rate, 4.

### Edman protein sequencing.

Edman protein sequencing was used to further characterize early fragments of native FtsZ degradation. To this end, 5 μM FtsZ protein was digested by ADEP2-ClpP as described above and subsequently separated by SDS-PAGE (12%), followed by semidry electroblotting on a polyvinylidene difluoride (PVDF) membrane (blot buffer was 50 mM sodium borate [pH 9.0], 0.1% SDS, and 20% methanol) for 4 h at 4°C. Transferred proteins were stained with Ponceau S solution (0.5% Ponceau S and 1% acetic acid in deionized water) for 6 min, and desired bands were excised. Automated Edman protein sequencing was performed by Genaxxon (Germany) using a Procise capillary-liquid chromatography (cLC) Edman sequencer (Applied Biosystems), including N-terminal degradation steps 1 to 5 with amino acid identification and verification.
