# Supplementary material for: Cell Division Protein FtsZ Is Unfolded for N-Terminal Degradation by Antibiotic-Activated ClpP
Source: mBio. 2020 Jun 30;11(3):e01006-20. doi: 10.1128/mBio.01006-20 (PMC7327170; doi:10.1128/mBio.01006-20)
Supplement: FIG S1 [file mBio.01006-20-sf001.pdf]

Supporting information

Cell division protein FtsZ is unfolded for N-terminal degradation by antibiotic-activated ClpP

Nadine Silber, Stefan Pan, Sina Schäkermann, Christian Mayer, Heike Brötz-Oesterhelt, Peter Sass

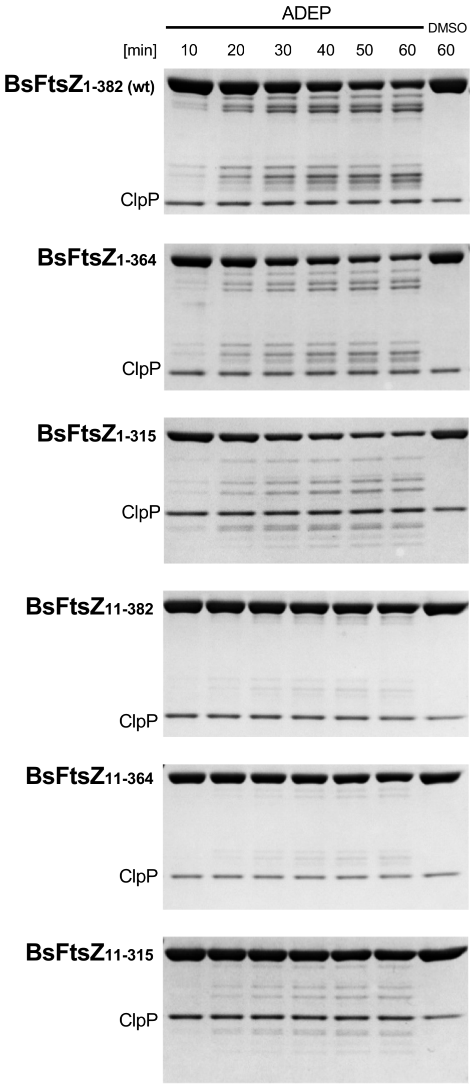

Figure S1:

Time-course of the degradation of FtsZ wild-type and mutant proteins by ADEP-ClpP.

Degradation of FtsZ wild-type and mutant proteins using low concentrations of ADEP and ClpP (1.5 µM ClpP; 1.5 µM ADEP) was followed over time. Samples were taken every 10 min over a time period of 60 min. DMSO was used as a control. The intensity of the ClpP protein band also serves as a loading control.
