# Supplementary material for: Cell Division Protein FtsZ Is Unfolded for N-Terminal Degradation by Antibiotic-Activated ClpP
Source: mBio. 2020 Jun 30;11(3):e01006-20. doi: 10.1128/mBio.01006-20 (PMC7327170; doi:10.1128/mBio.01006-20)
Supplement: FIG S2 [file mBio.01006-20-sf002.pdf]

Supporting information

Cell division protein FtsZ is unfolded for N-terminal degradation by antibiotic-activated ClpP

Nadine Silber, Stefan Pan, Sina Schäkermann, Christian Mayer, Heike Brötz-Oesterhelt, Peter Sass

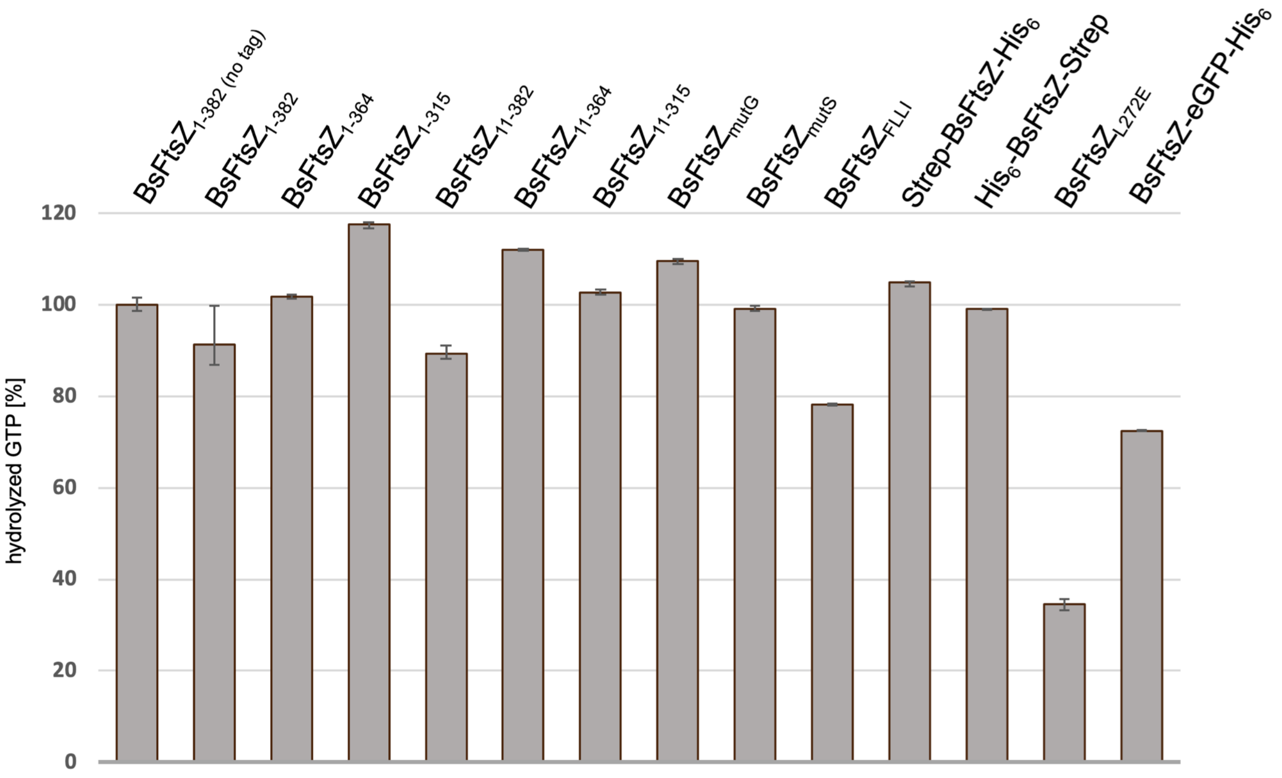

Figure S2:

GTPase activity assays of FtsZ wild-type and mutant proteins indicate their functionality.

Functionality of FtsZ was tested by comparing GTPase activities of full-length, wild-type proteins (BsFtsZ$_{1\text{-}382\ (no\ tag)}$ and BsFtsZ$_{1\text{-}382}$) and individual mutants. With the exception of BsFtsZ$_{1\text{-}382\ (no\ tag)}$, depicted proteins were expressed with a C-terminal His-6 tag. All mutants, except for BsFtsZ$_{L272E}$ as expected, retained GTPase activity mostly similar to wild-type level. Accordingly, an *E. coli* FtsZ$_{L272E}$ mutant was shown to bind nucleotides but was incapable to polymerize. Also for the BsFtsZ-eGFP fusion, GTPase activity of FtsZ was slightly reduced, which may be due to a disturbance by the eGFP fusion partner. In all assays, 10 µM of protein was used and GTP turnover was measured after 10 min. The mean of three biological replicates is indicated, error bars show highest and lowest values of the replicates. Wild-type, untagged BsFtsZ (BsFtsZ$_{1\text{-}382\ (no\ tag)}$) was set to 100%.
