# Supplementary material for: Cell Division Protein FtsZ Is Unfolded for N-Terminal Degradation by Antibiotic-Activated ClpP
Source: mBio. 2020 Jun 30;11(3):e01006-20. doi: 10.1128/mBio.01006-20 (PMC7327170; doi:10.1128/mBio.01006-20)
Supplement: FIG S3 [file mBio.01006-20-sf003.pdf]

Supporting information

Cell division protein FtsZ is unfolded for N-terminal degradation by antibiotic-activated ClpP

Nadine Silber, Stefan Pan, Sina Schäkermann, Christian Mayer, Heike Brötz-Oesterhelt, Peter Sass

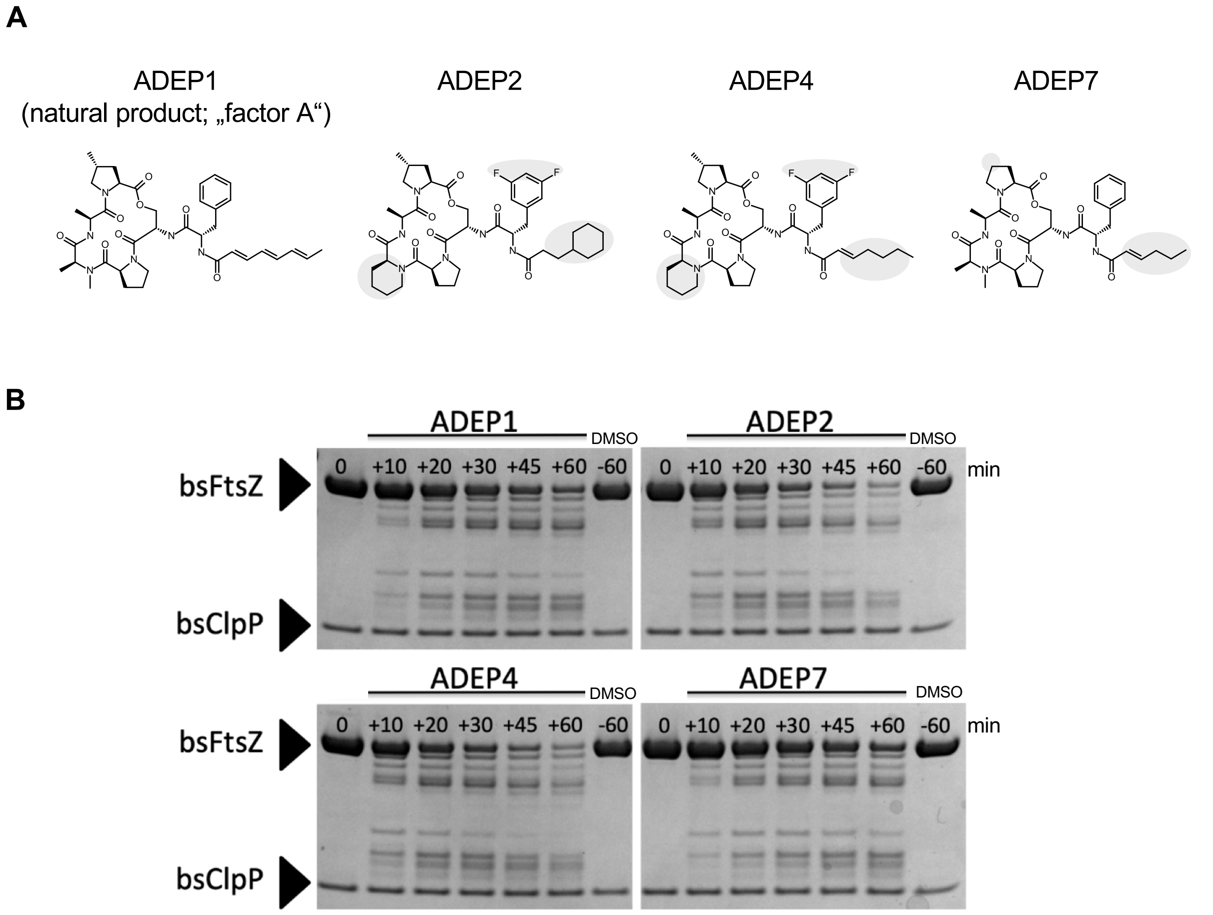

Figure S3:

ADEP derivatives differ in activating ClpP for the degradation of FtsZ.

(A) Structure of the natural product ADEP1 and its synthetic congeners ADEP2, 4, and 7. ADEP1 (“factor A”) is a natural product of *Streptomyces hawaiiensis* NRRL 15010 (4). The synthetic congeners have been reported previously (1). Highlighted regions indicate where the synthetic congeners deviate from the natural product ADEP1.

(B) SDS-PAGE analyses of *in vitro* ADEP-ClpP degradation assays using full-length $BsFtsZ_{1\text{-}382}$ and BsClpP proteins in combination with different ADEP derivatives. Here, ADEP2 and ADEP4 were most effective in activating BsClpP. DMSO was used as a control (“-60 min”). ADEP2 was selected for all subsequent experiments. All experiments were performed at least in triplicate, representative images are depicted.
