# Supplementary material for: Cell Division Protein FtsZ Is Unfolded for N-Terminal Degradation by Antibiotic-Activated ClpP
Source: mBio. 2020 Jun 30;11(3):e01006-20. doi: 10.1128/mBio.01006-20 (PMC7327170; doi:10.1128/mBio.01006-20)
Supplement: FIG S4 [file mBio.01006-20-sf004.pdf]

Supporting information

Cell division protein FtsZ is unfolded for N-terminal degradation by antibiotic-activated ClpP

Nadine Silber, Stefan Pan, Sina Schäkermann, Christian Mayer, Heike Brötz-Oesterhelt, Peter Sass

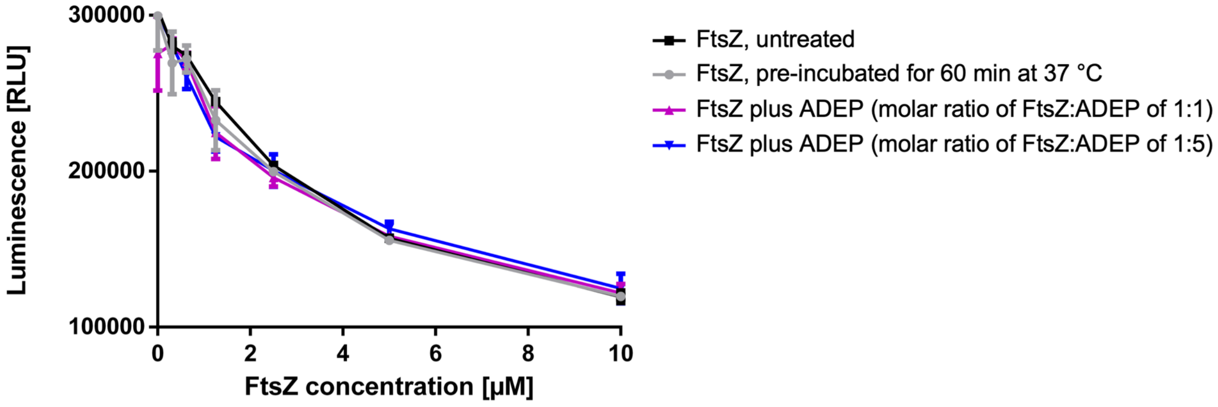

Figure S4:

ADEP does not interfere with FtsZ GTPase activity.

The target of ADEP is ClpP. To further exclude self-unfolding of FtsZ during incubation at 37 °C as well as off-target effects of ADEP on FtsZ activity in our *in vitro* assays, we tested the functionality of FtsZ under these conditions via GTPase activity assays. Non-hydrolysed GTP was read-out by conversion to ATP to fuel a luciferase reaction. Here, GTPase activity of FtsZ remained unaffected upon 60 min incubation at 37 °C. Hence, FtsZ does not turn unstable or functionally inactive during our *in vitro* assays. Furthermore, low or high concentrations of ADEP (molar ratio of FtsZ:ADEP2 of 1:1 or 1:5, respectively) did not affect GTPase activity, indicating that there are no off-target effects of ADEP on FtsZ to be expected. Of note, in the *in vitro* degradation assays of this study, the ADEP concentration never surpassed the molar ratio for FtsZ:ADEP2 of 1:1.6.
