# Supplementary material for: Cell Division Protein FtsZ Is Unfolded for N-Terminal Degradation by Antibiotic-Activated ClpP
Source: mBio. 2020 Jun 30;11(3):e01006-20. doi: 10.1128/mBio.01006-20 (PMC7327170; doi:10.1128/mBio.01006-20)
Supplement: FIG S5 [file mBio.01006-20-sf005.pdf]

Supporting information

Cell division protein FtsZ is unfolded for N-terminal degradation by antibiotic-activated ClpP

Nadine Silber, Stefan Pan, Sina Schäkermann, Christian Mayer, Heike Brötz-Oesterhelt, Peter Sass

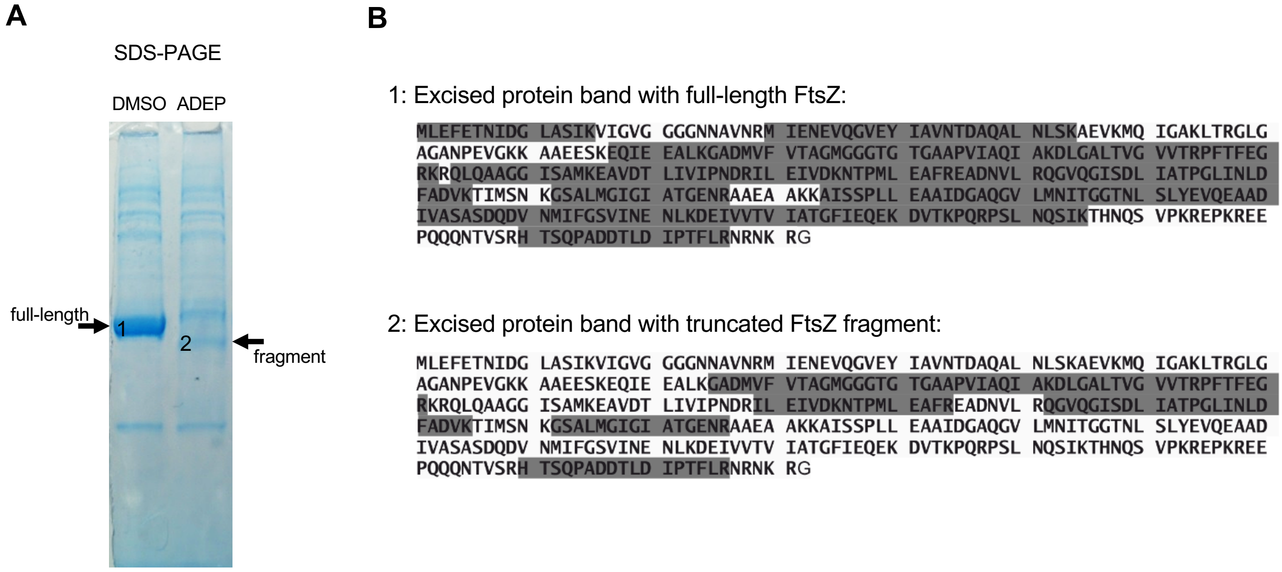

Figure S5:

ESI-MS of full-length and high-molecular weight fragments of FtsZ indicate N-terminal truncations following degradation by ADEP-ClpP.

(A) FtsZ was purified and incubated with ClpP in the presence of ADEP2 or DMSO (negative control) and subsequently separated by SDS-PAGE. Protein bands corresponding to FtsZ full-length protein in the control (1) and a fragment thereof appearing in the ADEP-treated sample (2) were excised from the gel, tryptically digested and subjected to orienting LC-ESI-MS studies. Low concentrations of ADEP/ClpP (1.5 µM ClpP monomer; 1.5 µM ADEP) were used.

(B) ESI-MS sequence coverages of FtsZ are highlighted in gray and show that the FtsZ fragment generated no N-terminal tryptic peptides compared to the full-length protein. Amino acid identification of the N-termini was then achieved using Edman protein sequencing (Fig. 4, main text).
