# Supplementary material for: Cell Division Protein FtsZ Is Unfolded for N-Terminal Degradation by Antibiotic-Activated ClpP
Source: mBio. 2020 Jun 30;11(3):e01006-20. doi: 10.1128/mBio.01006-20 (PMC7327170; doi:10.1128/mBio.01006-20)
Supplement: FIG S6 [file mBio.01006-20-sf006.pdf]

Supporting information

Cell division protein FtsZ is unfolded for N-terminal degradation by antibiotic-activated ClpP

Nadine Silber, Stefan Pan, Sina Schäkermann, Christian Mayer, Heike Brötz-Oesterhelt, Peter Sass

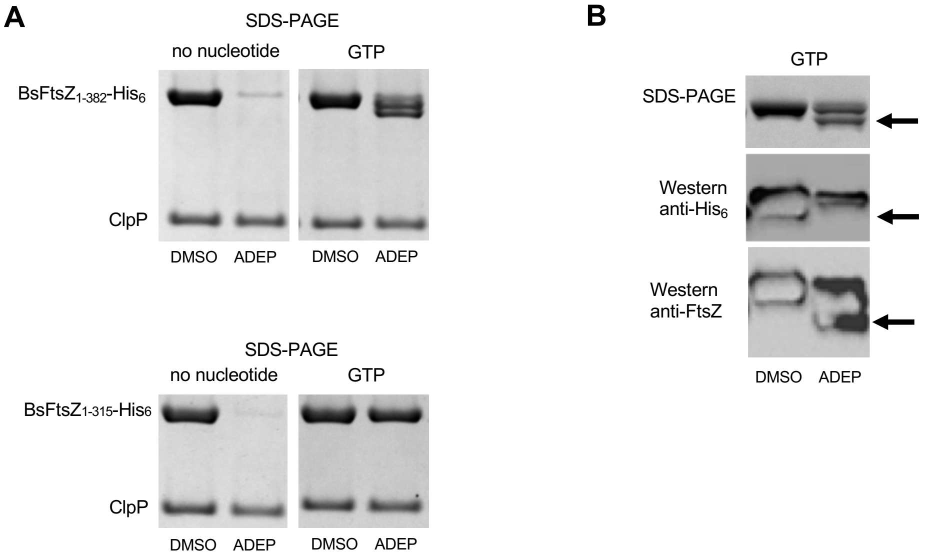

Figure S6:

The C-terminus of FtsZ is an additional target site at high concentrations of ADEP/ClpP.

(A) $FtsZ_{1\text{-}382}$ or $FtsZ_{1\text{-}315}$, both with attached C-terminal His_6-tags, were pre-incubated with or without GTP and subsequently used in ADEP-ClpP degradation assays with a high concentration of ADEP/ClpP (2.5 µM ClpP; 6.25 µM ADEP2). SDS-PAGE images show two distinct degradation products for $FtsZ_{1\text{-}382}$ after 120 min in the presence of ADEP-ClpP and GTP. Of note, no degradation bands were detected for $FtsZ_{1\text{-}315}$ in the presence of ADEP-ClpP and GTP. DMSO was used as a control.

(B) SDS-PAGE and corresponding Western blots using either anti-His_6 or anti-FtsZ antibodies show that the degradation products of $FtsZ_{1\text{-}382}$ lack the C-terminal His_6-tag, proving C-terminal attack by ADEP-ClpP.

Signals for anti-His_6 and anti-FtsZ antibodies were intentionally overexposed (resulting in white regions within the protein band) to also allow detection of weaker signals. Arrows mark the position of a C-terminal degradation product that could be detected by SDS-PAGE and with an anti-FtsZ antibody, but not with an anti-His6 antibody. DMSO was used as a control. All experiments were performed at least in triplicate, representative images are depicted.
