# Supplementary material for: Cell Division Protein FtsZ Is Unfolded for N-Terminal Degradation by Antibiotic-Activated ClpP
Source: mBio. 2020 Jun 30;11(3):e01006-20. doi: 10.1128/mBio.01006-20 (PMC7327170; doi:10.1128/mBio.01006-20)
Supplement: FIG S7 [file mBio.01006-20-sf007.pdf]

Supporting information

Cell division protein FtsZ is unfolded for N-terminal degradation by antibiotic-activated ClpP

Nadine Silber, Stefan Pan, Sina Schäkermann, Christian Mayer, Heike Brötz-Oesterhelt, Peter Sass

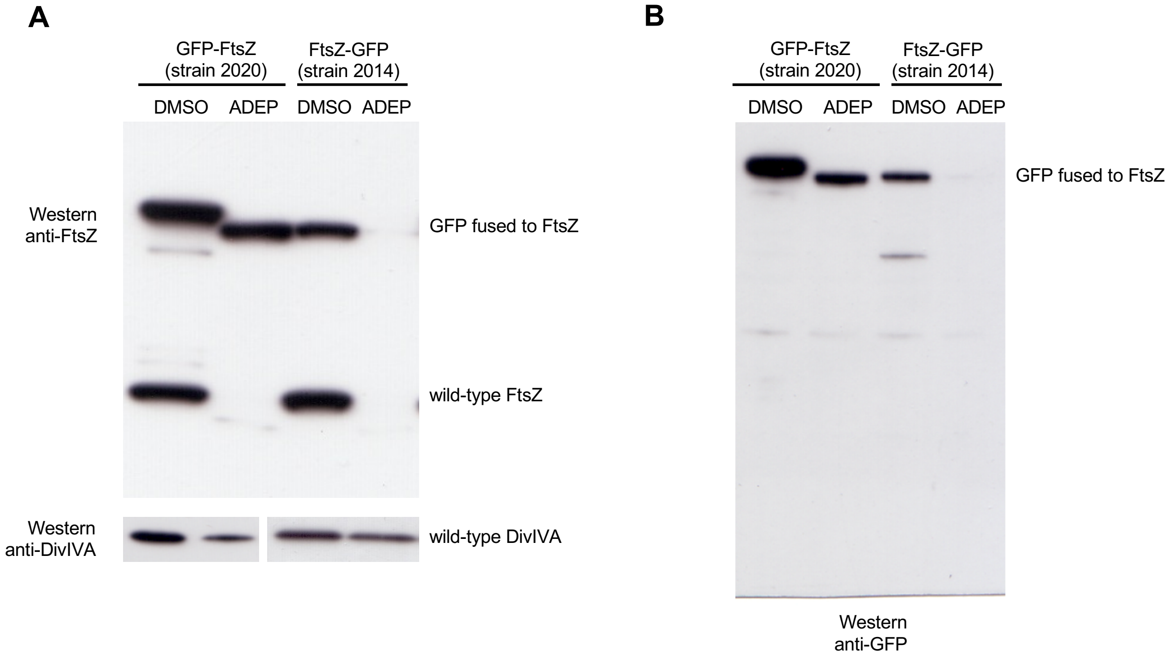

Figure S7:

ADEP-ClpP targets both termini of FtsZ at increased ADEP concentrations in whole cells.

Exponentially growing *B. subtilis* strains, which expressed both wild-type FtsZ as well as FtsZ mutant proteins attached to GFP (either fused to the N- or to the C-terminus of FtsZ) were treated for 60 minutes with either DMSO (negative control) or 0.5 µg/ml ADEP2, an antibiotic concentration that is well above the optimal filamentation concentration. Protein extracts were prepared and analysed via immunoblotting using anti-FtsZ (A) and anti-GFP antibodies (B). In accordance with our *in vitro* data of nucleotide-bound FtsZ, blocking the hydrophobic N-terminus of FtsZ with GFP only led to the truncation of the flexible C-terminus of FtsZ thereby generating a stable degradation product. On the contrary, when the C-terminus of FtsZ was blocked by GFP and the N-terminus remained accessible, FtsZ was fully degraded similar to wild-type FtsZ. Noteworthy, FtsZ-GFP runs sightly lower on SDS-PAGE than GFP-FtsZ which may be the result of the fusion of GFP to the long flexible C-terminus of FtsZ, probably providing more flexibility to the protein while running on SDS-PAGE compared to an N-terminal fusion. Detection of DivIVA protein using an anti-DivIVA antibody (A) served as a loading control. Further noteworty, we did not detect accumulating GFP fragments upon ADEP treatment of strain 2014 expressing the FtsZ-GFP fusion. Thus, we cannot exclude that GFP fragments of FtsZ-GFP may be further processed by either ADEP-ClpP or other proteases in the bacterial cell under these conditions. Images are representative of at least three independent experiments.
