# Supplementary material for: Cell Division Protein FtsZ Is Unfolded for N-Terminal Degradation by Antibiotic-Activated ClpP
Source: mBio. 2020 Jun 30;11(3):e01006-20. doi: 10.1128/mBio.01006-20 (PMC7327170; doi:10.1128/mBio.01006-20)
Supplement: TABLE S1 [file mBio.01006-20-st001.pdf]

Cell division protein FtsZ is unfolded for N-terminal degradation by antibiotic-activated ClpP
Nadine Silber, Stefan Pan, Sina Schäkermann, Christian Mayer, Heike Brötz-Oesterhelt, Peter Sass

S1 Table. Bacterial strains and plasmids

Strain /plasmid	Relevant characteristic(s) /genotype	Ref. /Source
Strains		
B. subtilis		
168	*trpC2*; wild type strain	(1)
2014	*trpC2 chrI::pJSIZΔpble (Pspac-ftsZ ble)* Ω*(Pxyl-ftsZ-gfpmut1 cat)*	(2)
2020	*trpC2 spc amyE::Pxyl-gfp-ftsZ*	(3)
S. aureus		
NCTC 8325	*rsbU, tcaR;* wild-type strain	NARSA
E. coli		
K-12 JM109	subcloning host	(4)
Bl21(DE3)	λDE3 lysogen; expression host	(5)
W3110 (pBS58)(pCXZ)	Strain W3110 carrying the plasmids pCXZ(Ptac-*ftsZ*bs, Amp^{R}) and pBS58 (*ftsQAZ*ec, Spc^{R})	(6)
Plasmids		
pClpP11	pQE70 (Qiagen) + ORF BSU34540 (*clpP*)	(7)
pET22b	vector for the expression of C-terminal His$_6$ fusion proteins	Novagen
pET11a	vector for the expression of native proteins	Novagen
pET22BΔ*pelB*	pET22b without *pelB*-leader tag, for the expression of C-terminal His$_6$ fusion proteins	(8)
pNP90	pET21d (Novagen) + ORF BSU15290 (*B. subtilis ftsZ*)	Hamoen lab
pETftsZsa	pET22BΔ*pelB* + ORF SAOUHSC_01150 (*S. aureus ftsZ*)	this study
pETclpPsa	pET22BΔ*pelB* + ORF SAOUHSC_00790 (*S. aureus clpP*)	this study
pETftsZbs$_{1-315}$	pET22BΔ*pelB* + ORF BSU15290 (*B. subtilis ftsZ*) comprising aa 1-315	this study
pETftsZbs$_{1-364}$	pET22BΔ*pelB* + ORF BSU15290 (*B. subtilis ftsZ*) comprising aa 1-364	this study
pETftsZbs$_{11-382}$	pET22BΔ*pelB* + ORF BSU15290 (*B. subtilis ftsZ*) comprising aa 11-382	this study
pETftsZbs$_{11-315}$	pET22b + ORF BSU15290 (*B. subtilis ftsZ*) comprising aa 11-315	this study
pETftsZbs$_{11-364}$	pET22b + ORF BSU15290 (*B. subtilis ftsZ*) comprising aa 11-364	this study
pETftsZbs$_{mutG}$	pET22b + ORF BSU15290 (*B. subtilis ftsZ*) carrying aa mutations L2G, F4G, I8G, L11G	this study
pETftsZbs$_{mutS}$	pET22b + ORF BSU15290 (*B. subtilis ftsZ*) carrying aa mutations L2S, F4S, I8S, L11S	this study
pETftsZbs$_{FLLI}$	pET22b + ORF BSU15290 (*B. subtilis ftsZ*) carrying aa mutations L2F, F4L, I8L, L11I	this study
pETftsZbs$_{L272E}$	pNP90 carrying aa mutation L272E in the *B. subtilis ftsZ* gene	this study
pETftsZbs-strep	pET11a + ORF BSU15290 (*B. subtilis ftsZ*) with C-terminal Strep-tag II	
pETstrep-ftsZ-his$_6$	pNP90 with additional N-terminal Strep-tag II	this study
pEThis$_6$-ftsZ-strep	pET11a + ORF BSU15290 (*B. subtilis ftsZ*) with attached N-terminal His$_6$-tag and C-terminal Strep-tag II	this study
pET22b-egfp	pET22b + *egfp* gene (UniProtKB ID C5MKY7)	this study
pET22b-NZ-egfp	pET22b + *egfp* gene (UniProtKB ID C5MKY7) carrying aa 1-10 of ORF BSU15290 (*B. subtilis* FtsZ$_{1-10}$) attached to the N-terminus of eGFP	this study
pDEST007 -strep-egfp	pDest007-eGFP-(Ec)-ssrA with deleted ssrA tag	(9), this study
pDEST007 -strep-egfp-NZ	pDEST007-strep-gfp carrying aa 1-10 of ORF BSU15290 (FtsZ$_{10-1}$) attached to the C-terminus of eGFP	this study
pETftsZbs-egfp$_{H6}$	pET22b + *egfp* gene (UniProtKB ID C5MKY7) with an His$_6$-tag fused to the C-terminus of *B. subtilis ftsZ* (ORF BSU15290)	this study
pET$_{H6}$egfp$_{H6}$	pET22b + *egfp* gene (UniProtKB ID C5MKY7) with an N- and C-terminal His$_6$-tag	this study
pSpx	pQE-60 (Qiagen) + ORF BSU11500 (*B. subtilis yjbD*)	(7)
pQE-NZ-Spx	pSpx carrying aa 1-10 of ORF BSU15290 (*B. subtilis* FtsZ$_{1-10}$) attached to the N-terminus of Spx	this study
pET11a-bsEF-Tu	pET11a + ORF BSU01130 (*B. subtilis tufA*)	this study
pET11a-bsPyk	pET11a + ORF BSU29180 (*B. subtilis pyk*)	this study
pET11a-bsFbaA	pET11a + ORF BSU37120 (*B. subtilis fbaA*)	this study

Abbreviations: aa, amino acids; ORF BSU, open reading frame of *Bacillus subtilis* 168 (genome accession number NC_000964), N-terminus, amino-terminus; C-terminus, carboxy-terminus; eGFP, enhanced green fluorescent protein; NZ, amino-acids 1-10 of BSU15290 (FtsZ$_{1-10}$); NARSA, Network on Antimicrobial Resistance in *Staphylococcus aureus*.

Table S1 references

1. Anagnostopoulos C, Spizizen J. 1961. Requirements for transformation of *Bacillus subtilis*. J Bacteriol 81:741-746.
2. Sievers J, Errington J. 2000. The *Bacillus subtilis* cell division protein FtsL localizes to sites of septation and interacts with DivIC. Mol Microbiol 36:846-855.
3. Stokes NR, Sievers J, Barker S, Bennett JM, Brown DR, Collins I, Errington VM, Foulger D, Hall M, Halsey R, Johnson H, Rose V, Thomaides HB, Haydon DJ, Czaplewski LG, Errington J. 2005. Novel inhibitors of bacterial cytokinesis identified by a cell-based antibiotic screening assay. J Biol Chem 280:39709-39715.
4. Yanisch-Perron C, Vieira J, Messing J. 1985. Improved M13 phage cloning vectors and host strains: nucleotide sequences of the M13mp18 and pUC19 vectors. Gene 33:103-119.
5. Studier FW, Moffatt BA. 1986. Use of bacteriophage T7 RNA polymerase to direct selective high-level expression of cloned genes. J Mol Biol 189:113-130.
6. Wang X, Lutkenhaus J. 1993. The FtsZ protein of *Bacillus subtilis* is localized at the division site and has GTPase activity that is dependent upon FtsZ concentration. Mol Microbiol 9:435-442.
7. Turgay K, Hahn J, Burghoorn J, Dubnau D. 1998. Competence in *Bacillus subtilis* is controlled by regulated proteolysis of a transcription factor. EMBO J 17:6730-6738.
8. Sass P, Bierbaum G. 2007. Lytic activity of recombinant bacteriophage phi11 and phi12 endolysins on whole cells and biofilms of *Staphylococcus aureus*. Appl Environ Microbiol 73:347-352.
9. Gersch M, Famulla K, Dahmen M, Gobl C, Malik I, Richter K, Korotkov VS, Sass P, Ruebsamen-Schaeff H, Madl T, Brötz-Oesterhelt H, Sieber SA. 2015. AAA+ chaperones and acyldepsipeptides activate the ClpP protease via conformational control. Nat Commun 6:6320.
