# Supplementary material for: Cell Division Protein FtsZ Is Unfolded for N-Terminal Degradation by Antibiotic-Activated ClpP
Source: mBio. 2020 Jun 30;11(3):e01006-20. doi: 10.1128/mBio.01006-20 (PMC7327170; doi:10.1128/mBio.01006-20)
Supplement: TABLE S2 [file mBio.01006-20-st002.pdf]

Supporting information

Cell division protein FtsZ is unfolded for N-terminal degradation by antibiotic-activated ClpP

Nadine Silber, Stefan Pan, Sina Schäkermann, Christian Mayer, Heike Brötz-Oesterhelt, Peter Sass

S2 Table. Primer used in this study.

Plasmid	Forward (F) / Reverse (R) oligo (5'-3' direction)	Template
pETftsZsa	F: tttccatggtagaatttgaacaaggatttaatc<br>R: aaactcgagacgtcttgttcttcttgaacg	*S. aureus* gDNA
pETclpPsa	F: Tttccatggatttaattcctacagttattgaaac<br>R: aaactcgagttttgtttcaggtaccatcacttc	*S. aureus* gDNA
pETftsZbs$_{1-315}$	F: tttccatggtggagttcgaaacaaacatagac<br>R: aaactcgagaaagccggttgcaatcactg	*B. subtilis* gDNA
pETftsZbs$_{1-364}$	F: tttccatggtggagttcgaaacaaacatagac<br>R: aaactcgagcggctgtgaagtatgacgg	*B. subtilis* gDNA
pETftsZbs$_{11-382}$	F: tttccatggcatcaattaaagtaatcggagtag<br>R: aaactcgaggccgcgtttattacggtttc	*B. subtilis* gDNA
pETftsZbs$_{11-315}$	F: tttccatggcatcaattaaagtaatcggagtag<br>R: aaactcgagaaagccggttgcaatcactg	*B. subtilis* gDNA
pETftsZbs$_{11-364}$	F: tttccatggcatcaattaaagtaatcggagtag<br>R: aaactcgagcggctgtgaagtatgacgg	*B. subtilis* gDNA
pETftsZbs$_{mutG}$	F: tttcatatgggagagggagaaacaaacggagacggcggagcatcaattaaagtaatcggagtag<br>R: aaactcgaggccgcgtttattacggtttc	*B. subtilis* gDNA
pETftsZbs$_{mutS}$	F: tttcatatgagcgagagcgaaacaaacagcgacggcagcgcatcaattaaagtaatcggagtag<br>R: aaactcgaggccgcgtttattacggtttc	*B. subtilis* gDNA
pETftsZbs$_{FLLI}$	F: tttcatatgttcgagttggaaacaaacttagacg gcatagcatcaattaaagtaatcggagtag<br>R: aaactcgaggccgcgtttattacggtttc	*B. subtilis* gDNA
pETftsZbs$_{L272E}$	F: gaacaaacctcagcgaatatgaggttcag<br>R: ctgaacctcatattcgctgaggtttgttc	pNP90
pETstrep-ftsZ-his$_6$	F: P-tggagccacccgcagttcgaaaaggtggagttcga aacaaacatag<br>R: P-catggtatatctccttcttaaag	pNP90
pETftsZbs-strep	F: aaacatatgttggagttcgaaacaaac<br>R: aaaggatcccttttcgaactgcgggtggctccactcgaggccgcgtttattacg	*B. subtilis* gDNA
pEThis$_6$-ftsZ-strep	F: P-gtggtggtgcatatgtatatctccttcttaaagttaaac<br>R: P-caccaccacttggagttcgaaacaaacatagac	pETftsZbs-strep
pET22b-egfp	F: aaacatatggtgagcaagggcgaggag<br>R: aaactcgaggctgcccagctcgtcc	pDest007-eGFP-(Ec)-ssrA
pET22b-NZ-egfp	F: P-tttcgaactccaacatatgtatatctccttcttaaagttaaac<br>R: P-caaacatagacggcttagtgagcaagggcgagg	pET22b-egfp
pDEST007<br>-strep-egfp-NZ	F: P-acagagttcgagttgtaacacccagctttcttgtacaaagtgg<br>R: P-attgatgtctccaagcttgtacagctcgtccatgccgag	pDest007-eGFP-(Ec)-ssrA
pDEST007<br>-strep-egfp	F: tttgaattccacccagctttcttgtacaaagtgg<br>R: tttgaattcttacttgtacagctcgtccatgcc	pDEST007-strep-egfp-NZ
pETftsZbs-egfp$_{H6}$	F: ggctcaggaagcggctcaggctccgtgagcaagggcgaggag<br>R: atgtatatctccttcttaaagttaaacaaaattatttctagagg<br>F: tttaagaaggagatatacatatgttggagttcgaaacaaac<br>R: ggagcctgagccgcttcctgagccgccgcgtttattacggtttc	*B. subtilis* gDNA/ pET22b-egfp,<br>Gibson assembly
pET22b$_{H6}$egfp$_{H6}$	F: ttttcat**atg**caccaccaccaccaccacgtgagcaagggcgagg<br>R: aaaacatatgtatatctccttcttaaagttaaac	pET22b-egfp
pQE-NZ-Spx	F: P-caaacatagacggcttagttacactatacacatcaccaagc<br>R: P-tttcgaactccaacatggttaatttctcctctttaatgaattc	pSpx
pET11a-bsEF-Tu	F: aaacatatggctaaagaaaaattcgacc<br>R: aaaggatccttactcagtgattgtagaaacaacg	*B. subtilis* gDNA
pET11a-bsPyk	F: tcgggctttgttagcagccgttaaagaacgctcgcacg<br>R: taagaaggagatatacatatatgagaaaaattgtttgtacc<br>F: atatgtatatctccttcttaaagttaaac<br>R: cggctgctaacaaagccc	*B. subtilis* gDNA Gibson assembly
pET11a-bsFbaA	F: aaacatatgcctttagtttctatgacgg<br>R: aaaggatccttaagcttggtttgaagaacc	*B. subtilis* gDNA

Restriction sites are underlined. Linker regions, which were inserted between FtsZ and eGFP protein fusions, are highlighted in grey. Abbreviations: gDNA, genomic DNA; P-, 5' phosphorylated; eGFP, enhanced green fluorescent protein; NZ, amino-acids 1-10 of BSU15290 ($FtsZ_{1-10}$).
