# Supplementary material for: Cell Division Protein FtsZ Is Unfolded for N-Terminal Degradation by Antibiotic-Activated ClpP
Source: mBio. 2020 Jun 30;11(3):e01006-20. doi: 10.1128/mBio.01006-20 (PMC7327170; doi:10.1128/mBio.01006-20)
Supplement: FIG S8 [file mBio.01006-20-sf008.pdf]

Supporting information

Cell division protein FtsZ is unfolded for N-terminal degradation by antibiotic-activated ClpP

Nadine Silber, Stefan Pan, Sina Schäkermann, Christian Mayer, Heike Brötz-Oesterhelt, Peter Sass

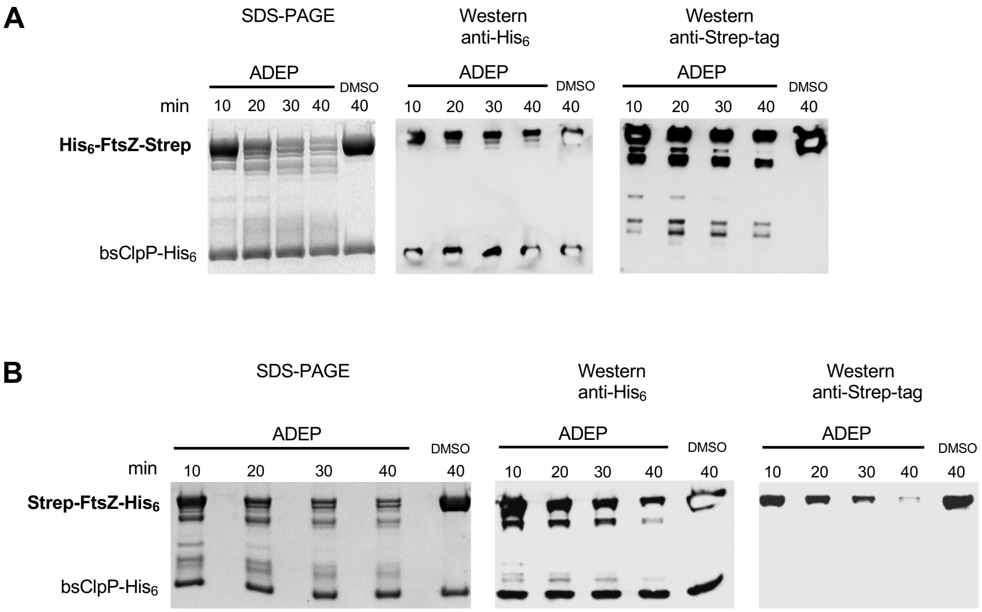

Figure S8:

Streptavidin or His_6 purification tags do not alter the degradation preference.

ADEP-ClpP degradation assays (high ADEP/ClpP concentration: 2.5 µM ClpP; 6.25 µM ADEP) using full-length $BsFtsZ_{1-382}$ proteins with a His_6-tag fused to the N-terminus and a Strep-tag attached to the C-terminus of FtsZ (A; His_6-FtsZ-Strep) or *vice versa* (B; Strep-FtsZ-His_6). DMSO was used as a control. Immunoblots were generated using anti-Strep or anti-His_6 specific antibodies as indicated. The ladder of bands that appeared as the typical degradation pattern for FtsZ can only be detected with tags attached to the C-terminus, confirming preferential targeting of the N-terminus by ADEP-ClpP. Furthermore the data shows, that these protein tags do not notably alter degradation preference of ADEP-ClpP. Signals for anti-His_6 and anti-strep antibodies are intentionally overexposed (resulting in white regions within the protein band) to also allow detection of weaker signals in the area of emerging degradation products. All experiments were performed at least in triplicate, representative images are depicted.
